# Rab33b-exocyst interaction mediates localized secretion for focal adhesion turnover and cell migration

**DOI:** 10.1016/j.isci.2022.104250

**Published:** 2022-04-14

**Authors:** Synne Arstad Bjørnestad, Noemi Antonella Guadagno, Ingrid Kjos, Cinzia Progida

**Affiliations:** 1 Department of Biosciences, University of Oslo, 0316 Oslo, Norway

**Keywords:** Cell biology, Organizational aspects of cell biology, Functional aspects of cell biology

## Abstract

Rab proteins are well known regulators of intracellular trafficking; however, more and more studies point to their function also in other cellular processes, including cell migration. In this work, we have performed an siRNA screen to identify Rab proteins that influence cell migration. The screen revealed Rab33b as the strongest candidate that affected cell motility. Rab33b has been previously reported to localize at the Golgi apparatus to regulate Golgi-to-ER retrograde trafficking and Golgi homeostasis. We revealed that Rab33b also mediates post-Golgi transport to the plasma membrane. We further identified Exoc6, a subunit of the exocyst complex, as an interactor of Rab33b. Moreover, our data indicate that Rab33b regulates focal adhesion dynamics by modulating the delivery of cargo such as integrins to focal adhesions. Altogether, our results demonstrate a role for Rab33b in cell migration by regulating the delivery of integrins to focal adhesions through the interaction with Exoc6.

## Highlights

• RNAi screen reveals a role for Rab33b in cell migration
• Rab33b influences focal adhesion dynamics
• Rab33b interacts with the exocyst subunit Exoc6
• Rab33b together with Exoc6 mediates the delivery of β1 integrin to adhesion points

## Graphical abstract

**Figure:**
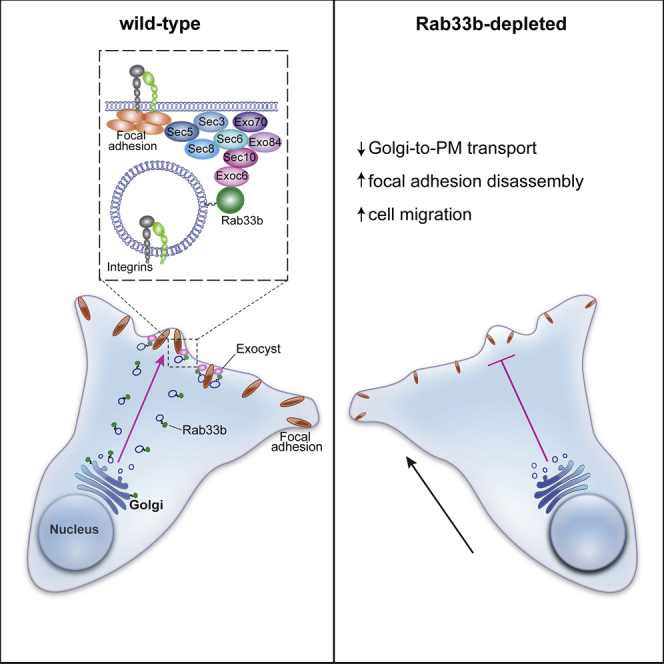

## Introduction

The Rab family of small GTPases is the largest family in the Ras superfamily with more than 60 members identified in humans (Guadagno and Progida, 2019). Rab proteins function as master regulators of intracellular trafficking alternating between an active GTP-bound state and an inactive GDP-bound state, and the interchange between these two states regulates their recruitment to membranes and interaction with a variety of different effectors (Muller and Goody, 2018). The Rab effectors include sorting adaptors for sorting of cargo and vesicle formation, phosphoinositide kinases and phosphatases, motor proteins for transport along the cytoskeleton, and tethering factors for tethering of transport vesicles to their target membranes (Zhen and Stenmark, 2015).

More recently, it has been revealed that Rab proteins, in addition to being important for intracellular transport, also have other functions. Indeed, they are involved in the regulation of mitotic spindle and abscission during cell division (Fremont and Echard, 2018; Gibieza and Prekeris, 2017), nutrient sensing and signaling (Lanzetti et al., 2004; Yang et al., 2016), modulation of the activity of autophagic regulators (Ao et al., 2014; Kjos et al., 2017), and regulation of cytoskeleton dynamics (Borg et al., 2014; Kjos et al., 2018; Klinkert and Echard, 2016; Vestre et al., 2019). Furthermore, several Rabs are involved in the process of cell migration. Rab5 was the first Rab shown to influence cell migration by promoting lamellipodia formation (Spaargaren and Bos, 1999), and since then several other studies have provided further evidence for Rab proteins in cell migration (Borg et al., 2014; Caswell et al., 2007; Margiotta et al., 2017; Palamidessi et al., 2008; Vestre et al., 2019, 2021; Wang et al., 2010). Rabs regulate cell migration in different ways. Rab5, for example, controls endocytosis, which is important for the localized activation of Rac (Palamidessi et al., 2008). Several Rab proteins, including Rab8, Rab6, and Rab7b, can indeed influence cytoskeleton dynamics by modulating the activity of members of the Rho family of small GTPases (Borg et al., 2014; Bravo-Cordero et al., 2016; Kjos et al., 2018; Vestre et al., 2019). Other Rabs take part in integrin recycling (Sahgal et al., 2019; Wang et al., 2010) or in focal adhesion turnover (Guadagno et al., 2020; Mendoza et al., 2013). Given the involvement of these small GTPases in cell motility, it is not surprising that their abnormal regulation is often associated with migratory ability and invasiveness in different types of cancer (Diaz et al., 2014; Guadagno and Progida, 2019; von Thun et al., 2012; Wang et al., 2016).

In this study, we performed an siRNA screen to identify Rab proteins involved in cell migration. From the primary screen, we selected the small GTPase Rab33b. Rab33b was the candidate showing the most dramatic and consistent phenotype as its depletion strongly promotes cell migration. Nothing is known regarding the involvement of Rab33b in the process of cell migration. Therefore, to identify the mechanism used by this small GTPase to affect cell motility, we searched for potential interactors by yeast two-hybrid screening, and identified the exocyst subunit Exoc6 (also known as Sec15) as a putativeRab33b interactor. The interaction was confirmed by co-immunoprecipitation and pulldown assays, and the two proteins were detected together on secretory vesicles in proximity of focal adhesion sites. Moreover, we showed that Rab33b, together with Exoc6, delivers integrins to focal adhesions, and that its depletion inhibits the transport along the secretory pathway, prevents Exoc6 recruitment to vesicles, and promotes focal adhesion disassembly. Altogether, our results indicate that Rab33b, via the interaction with Exoc6, influences cell migration by regulating focal adhesion turnover through the delivery of integrins to adhesion sites.

## Results

### An siRNA screen identifies Rab33b as a negative regulator of cell migration

**Figure 1:**
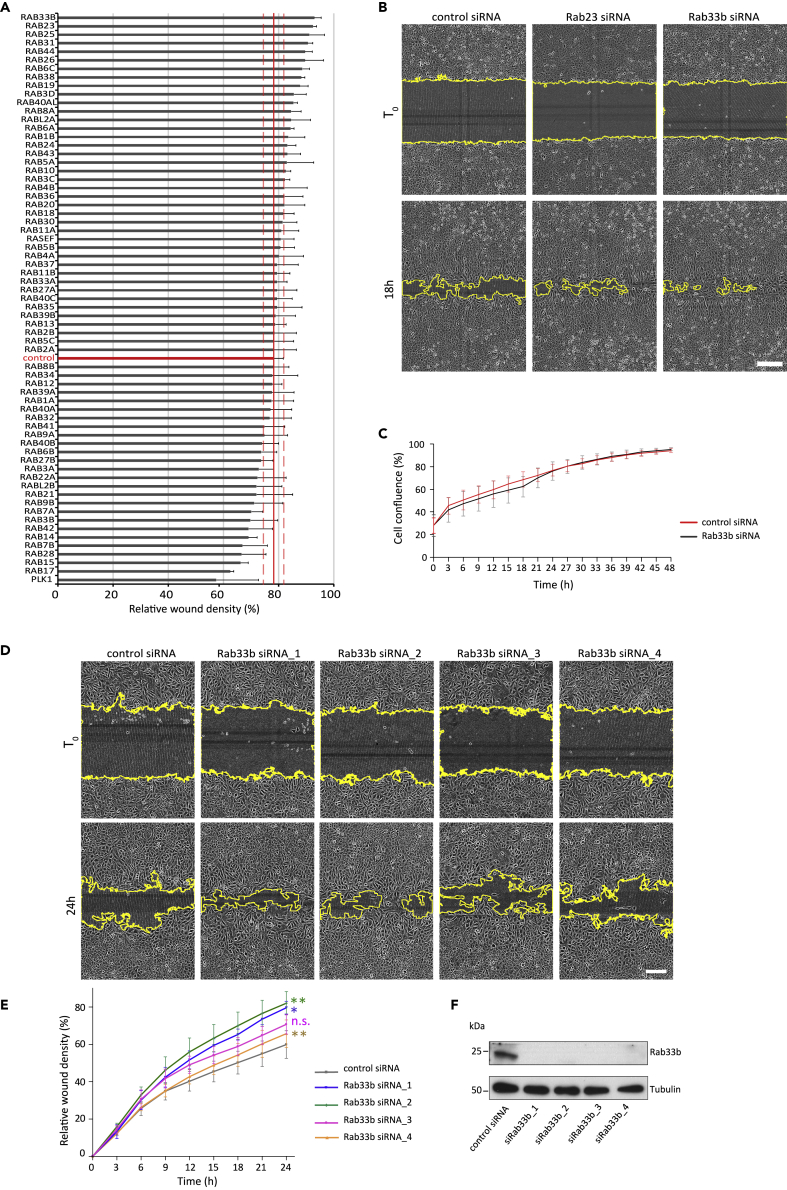
siRNA screen to identify Rab proteins affecting cell migration (A) U2OS cells transfected with control siRNA or pools of four different siRNAs against human Rabs were grown to confluency, scratch-wounded, and imaged every 3 h. The graph shows the relative wound density at 18 h after wounding represented as mean ± SEM of four independent experiments. The red solid line indicates the relative wound density (%) and the dashed red lines the boundaries of the SEM for cells transfected with control siRNA. (B) Representative images are shown for time 0 and 18 h after wounding for the two Rabs whose depletion had the strongest effect on wound closure. Scale bar: 200 μm. (C) U2OS cells transfected with control siRNA or a pool of four different siRNAs targeting Rab33b were imaged for 48 h. The rate of proliferation (measured as percentage of cell confluence) over time is shown as mean ± SEM of three independent experiments. (D) U2OS cells were transfected with control siRNA or each of the different individual four siRNAs present in the pool targeting Rab33b (Rab33b siRNA_1, siRNA_2, siRNA_3, or siRNA_4), grown to confluency, scratch-wounded, and imaged every 3 h. Representative images for time 0 and 24 h after wounding are shown. Scale bar: 200 μm. (E) Graph showing the relative wound density (%) over time for each sample in (d). The graph represents the mean ± SEM of a minimum of three independent experiments. ∗p < 0.05, ∗∗p < 0.01, n.s. not significant, for t = 24h (two-tailed paired Student′s t-test). (F) Lysates from U2OS cells transfected with control siRNA, or with each of the different individual four siRNAs present in the pool targeting Rab33b were subjected to Western blot analysis with the indicated antibodies.

To identify novel Rab proteins that influence cell migration, we performed an siRNA screen based on a wound healing assay. U2OS cells were transfected with an siRNA library consisting of siRNA pools targeting a total of 65 different Rab proteins in addition to a non-targeting control siRNA, and, as control for successful reverse transfection, siRNAs against the polo like kinase 1 (PLK1), a known regulator of cell division (Bruinsma et al., 2012), and grown for 48 h. The cell monolayer was scratch-wounded to make a cell-free zone (wound) and wound healing was evaluated after 18 h. The analysis of the relative wound density revealed that depletion of several Rabs influenced cell migration compared to cells transfected with control siRNA (Figure 1A). Among the hits, Rab33b was the candidate whose depletion more severely increased wound closure (Figures 1A–1B). To exclude that this effect was caused by an increased cell proliferation, we compared the proliferation between control cells and cells silenced for Rab33b. The results showed that silencing of Rab33b did not affect cell proliferation (Figure 1C), suggesting that Rab33b is indeed a regulator of cell migration. However, because the primary screen was performed using pools consisting of four different oligos for each target protein, we next validated separately the four siRNAs for Rab33b. From the deconvolution, all the four siRNAs resulted in increased cell migration, although to different extent, with siRNA_1 and siRNA_2 having the strongest effect, consistent with the results from the primary screen (Figures 1D–1F).

**Figure 2:**
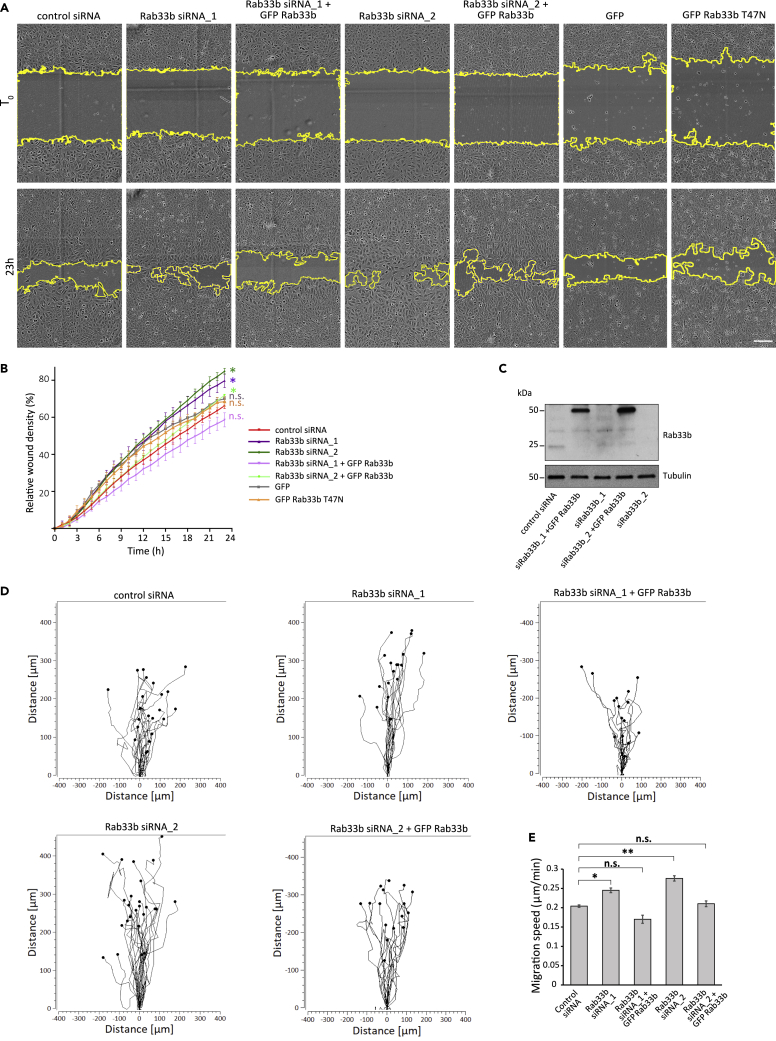
Rab33b depletion promotes cell migration (A) U2OS cells transfected with either control siRNA, Rab33b siRNA_1, Rab33b siRNA_2, treated with the same siRNAs against Rab33b and afterwards transfected with GFP-Rab33b or transiently transfected with GFP, or GFP-Rab33b T47N, were scratch-wounded and imaged every 3 h. Representative images for time 0 and 23 h after wounding are shown. Scale bar: 200 μm. (B) Graph showing relative wound density (%) for each sample in (a) over time. The graph represents the mean ± SEM of a minimum of three independent experiments. ∗p < 0.05, n.s., not significant, for t = 24h (two-tailed paired Student′s t-test). (C) Cell lysates from cells treated with control siRNA, Rab33b siRNA_1, Rab33b siRNA_2, or treated with the same siRNAs against Rab33b and afterwards transfected with GFP-Rab33b were subjected to Western blot analysis with antibodies against Rab33b and tubulin (as loading control). (D) Representative track plots of the single-cell distances of migration for cells transfected with control siRNA, Rab33b siRNA_1, Rab33b siRNA_2, or treated with the same siRNAs against Rab33b and afterwards transfected with GFP-Rab33b. Individual tracks are shown so that each starts at the origin (distance 0). (E) Quantification of the mean ± SEM of the single cell speed from at least three independent experiments. n > 30 cells per condition and per experiment. ∗p < 0.05, ∗∗p < 0.01, n.s., not significant (two-tailed paired Student′s t-test). See also Figures S1A–S1B

As the results from the oligo deconvolution confirmed that Rab33b silencing promotes wound closure, we selected the two siRNAs with strongest effect (Rab33b siRNA_1 and Rab33b siRNA_2) for follow-up studies. First, we evaluated whether the re-expression of Rab33b in U2OS cells depleted for this small GTPase rescued the effect on migration at levels similar to the control. The results showed indeed that transfection with GFP-Rab33b in cells silenced with either Rab33b siRNA_1 or Rab33b siRNA_2 was sufficient to rescue the rate of wound closure (Figures 2A–2C), further validating the specificity of the Rab33b siRNAs and of the migration defects upon Rab33b knockdown.

Having verified that the increase in cell migration upon Rab33b silencing is specific, we next investigated whether it is caused by an increase in single cell velocity. The tracking of single cells over time showed indeed that the velocity increased by over 20% for cells silenced with either Rab33b siRNA_1 or Rab33b siRNA_2 compared to control cells, and that the re-expression of Rab33b in the silenced cells restored the single cell speed to the levels of control cells (Figures 2D–2E). We finally validated the effect of Rab33b depletion on cell migration in a different cell line. Similar to the U2OS osteosarcoma cell line, retinal pigment epithelial-1 (RPE-1) cells silenced with either Rab33b siRNA_1 or Rab33b siRNA_2 also showed a tendency to increase cell migration (Figures S1A–S1B).

### Rab33b influences focal adhesion dynamics

**Figure 3:**
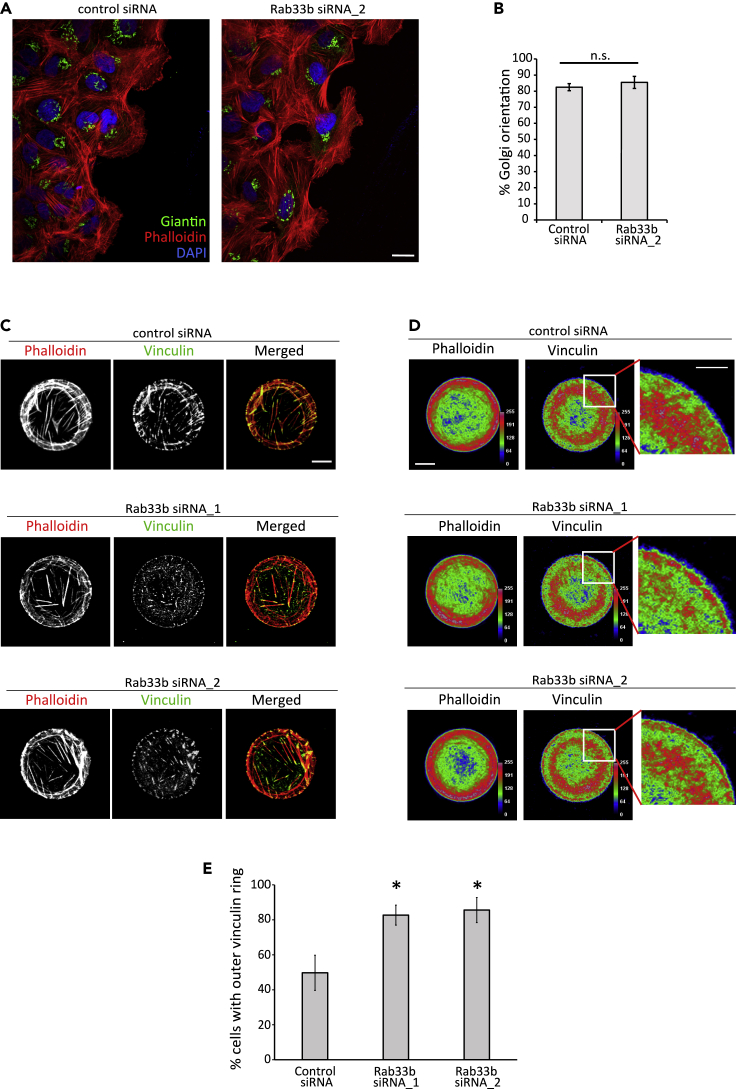
Silencing of Rab33b influences focal adhesions but not Golgi orientation (A) U2OS cells treated with siRNA control or Rab33b siRNA_2 were grown to confluency, scratch-wounded, and fixed after 6 h. Cells were immunostained with an antibody against giantin. Actin cytoskeleton was labeled with rhodamine-conjugated phalloidin and nuclei with DAPI. Scale bar: 10 μm. (B) Quantification of the percentage of cells having the Golgi apparatus located between the nucleus and the leading edge. The graph shows the mean ± SEM; n > 164 cells from four independent experiments. n.s., not significant (two-tailed paired Student′s t-test). (C) U2OS cells treated with siRNA control, Rab33b siRNA_1, or Rab33b siRNA_2, were plated onto fibronectin-coated disc-shaped micropatterns and left to adhere for 3.5 h before fixation and staining with rhodamine-conjugated phalloidin and an antibody against vinculin. Scale bar: 10 μm. (D) Color-coded map of the actin and vinculin distribution for U2OS cells treated with siRNA control or Rab33b siRNAs. The images were obtained by using the CellRef macro on averaged Z-projection images from aligned single stacks. The color scale is a color map of intensity generated by applying the Rainbow RGB LUT to the normalized mean cell (8-bit image). The color “red” indicates high intensity and the color “blue” indicates low intensity. Scale bar: 10 μm; inset: 5 μm. (E) Quantification of the percentage of cells having a vinculin ring at the cell periphery. The graph shows the mean ± SEM; n > 150 cells from three independent experiments. ∗p < 0.05 (two-tailed paired Student′s t-test). See also Figures S1C–S1D.

Rab33b is reported to be localized at the Golgi apparatus (Zheng et al., 1998), where it regulates Golgi-to-ER retrograde trafficking and Golgi homeostasis/organization (Starr et al., 2010; Valsdottir et al., 2001). As the Golgi apparatus reorients in migrating cells from a random perinuclear position to the area between the nucleus and the leading edge (Bisel et al., 2008; Kupfer et al., 1982), we next investigated whether Rab33b influences cell polarization in migrating cells. U2OS cells transfected with siRNA control or siRNAs against Rab33b were subjected to a wound healing assay and the orientation of the Golgi in migrating cells was analyzed by confocal microscopy. Quantification of Golgi reorientation in cells migrating toward the wound showed no effect upon Rab33b depletion (Figures 3A–3B).

**Figure 4:**
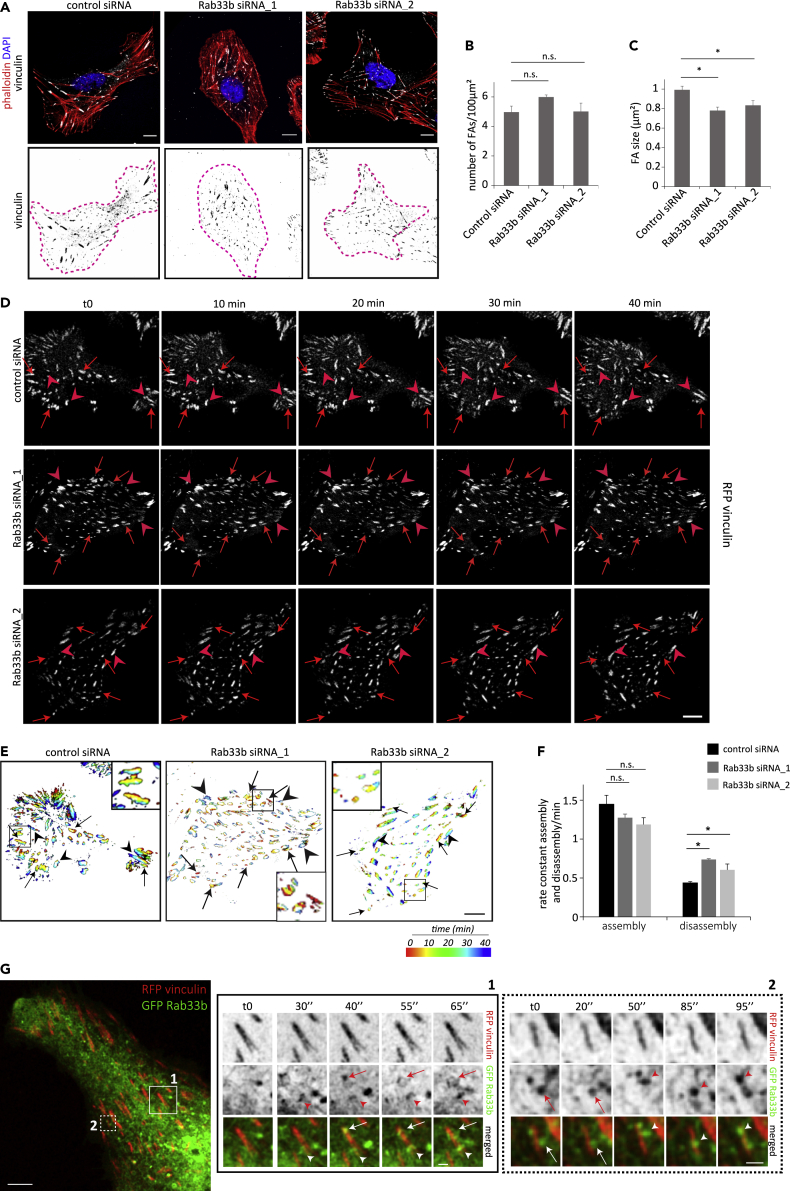
Rab33b is involved in the regulation of focal adhesion dynamics (A) U2OS cells silenced with control siRNA, Rab33b siRNA_1, or Rab33b siRNA_2, were fixed and stained with DAPI, rhodamine-conjugated phalloidin, and an antibody against vinculin. Scale bar: 5 μm. (B–C) Quantification of FA number per 100 μm^2^ cell area (b) and size (c). The graphs represent the mean ± SEM for three independent experiments (n > 90 cells). ∗p < 0.05, n.s., not significant (two-tailed paired Student′s t-test). (D) Control or Rab33b-depleted cells transfected with RFP-vinculin were imaged every 10 min for 40 min. Arrows show FA disassembly, and arrowheads show FA assembly. Scale bar: 10 μm. (E) Rainbow color representation of FA assembly and disassembly over time from cells shown in panel (d). Each time point is shown in a different color, as indicated in the bar. Insets show magnifications of the boxed areas. Scale bar: 10 μm. (F) Quantification of assembly and disassembly rates of FAs. The assembly and disassembly rate is shown as percentage of focal adhesion formation or disassembly per minute. The values represent the mean ± SEM from three independent experiments, in which 15 FAs were analyzed per cell (n > 5), per condition, and per experiment. ∗p < 0.05, n.s., not significant (two-tailed paired Student′s t-test). (G) Live-cell imaging of U2OS cells co-transfected with GFP-Rab33b (green) and RFP-vinculin (red). Cells were imaged every 5 s with a Zeiss LSM880 confocal microscope. Magnifications of the boxed areas in the side panels show Rab33b-positive vesicles moving to focal adhesion sites. Scale bar: 5μm, inset: 1μm. See also Figure S2 and Video S1. Rab33b-positive vesicles are delivered to FAs, Live-cell imaging of U2OS cells co-transfected with GFP-Rab33b (green) and vinculin-RFP (red). Magnification of the boxed area 1 in Figure 4g is shown and illustrates two examples of Rab33b-positive vesicles contacting focal adhesions. Cells were imaged every 5 s using a spinning disk confocal microscope. Related to Figure 4., Video S2. Rab33b-positive vesicles are delivered to FAs,Live-cell imaging of U2OS cells co-transfected with GFP-Rab33b (green) and vinculin-RFP (red). Magnifications of the boxed area 2 in Figure 4g is shown and illustrate two examples of Rab33b-positive vesicles contacting focal adhesions. Cells were imaged every 5 s using a spinning disk confocal microscope. Related to Figure 4., Video S3. Rab33b-positive vesicles are delivered to growing FAs during membrane protrusion,U2OS cells transiently transfected with GFP-Rab33b (green) and vinculin-RFP (red) were imaged every 30 s using a TIRF microscope with a penetration depth of 90 nm. The movie shows the recruitment of GFP-Rab33b-positive vesicles during membrane protrusion. Related to Figure 4..

To further confirm that Rab33b depletion does not affect the Golgi apparatus nor actin organization, we took advantage of adhesive micropatterns, which force cells to adapt to the adhesive-patterned shapes (Thery, 2010). This method allows an easier comparison and the identification of possible effects on cytoskeleton or Golgi architecture caused by Rab33b knockdown. While we could not detect any major alteration in the Golgi apparatus and actin distribution or organization, focal adhesion (FA) distribution, revealed by vinculin immunolabeling, was affected by Rab33b depletion (Figures 3C–3D and S1C–S1D). A ring of small adhesions was present at the periphery of the cells plated on disc-shaped patterns in over 80% of the cells knocked down for Rab33b, whereas less than the half of control cells presented a similar organization (Figure 3E). We therefore wondered whether Rab33b is involved in the regulation of FAs and analyzed FA size and number in cells silenced for Rab33b. While the number of FAs in cells knocked down for Rab33b was similar to the number of FAs in control cells, FA size decreased about 20% compared to the size in control cells (Figures 4A–4C). This led us to hypothesize that Rab33b might regulate FA dynamics. To verify this, we measured FA turnover in live cells transfected with RFP-vinculin. FA assembly and disassembly rates were calculated as the percentage of FAs that assembled or disassembled per minute (Guadagno et al., 2020). In agreement with the data on FA size, FA dynamics was affected by the knockdown of Rab33b. Indeed, the assembly rate was over 20% lower in silenced cells compared with controls, and the disassembly rate was 40% higher (Figures 4D–4F), indicating that Rab33b is required for the regulation of FA dynamics.

We then investigated if this occurs through a physical association between Rab33b and FAs by analyzing whether Rab33b-positive vesicles are delivered to FAs using live-cell imaging. Intriguingly, live imaging experiments revealed that GFP-Rab33b vesicles are delivered to FAs (Figure 4G, and Video S1, and S2), pointing to the existence of a Rab33b-mediated pathway directed toward FAs for the regulation of FA turnover. Total internal reflection fluorescence (TIRF) microscopy analysis further revealed that this process is even more evident during protrusion formation. An increase of Rab33b-positive vesicles occurs indeed concomitant to the formation of membrane protrusion and to the faster FA turnover (Figure S2 and Video S3).

Document S1. Figures S1–S5 and Table S1

Video S1. Rab33b-positive vesicles are delivered to FAs, Live-cell imaging of U2OS cells co-transfected with GFP-Rab33b (green) and vinculin-RFP (red). Magnification of the boxed area 1 in Figure 4g is shown and illustrates two examples of Rab33b-positive vesicles contacting focal adhesions. Cells were imaged every 5 s using a spinning disk confocal microscope. Related to Figure 4.

Video S2. Rab33b-positive vesicles are delivered to FAs,Live-cell imaging of U2OS cells co-transfected with GFP-Rab33b (green) and vinculin-RFP (red). Magnifications of the boxed area 2 in Figure 4g is shown and illustrate two examples of Rab33b-positive vesicles contacting focal adhesions. Cells were imaged every 5 s using a spinning disk confocal microscope. Related to Figure 4.

### The exocyst subunit Exoc6 is an interactor of Rab33b

**Figure 5:**
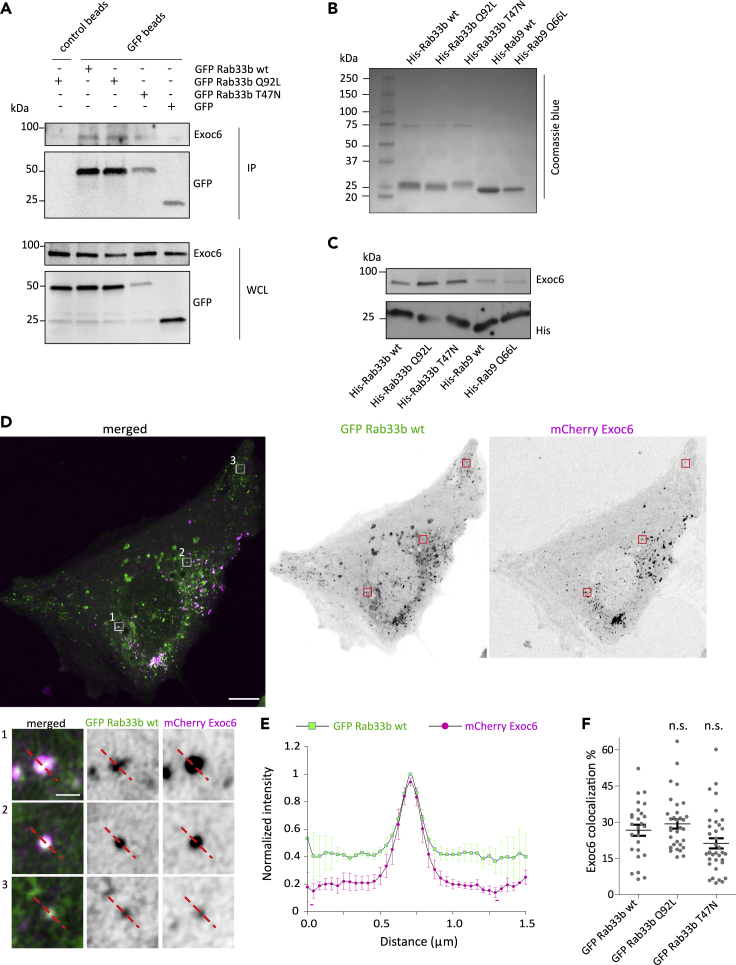
Exoc6 is a Rab33b interactor (A) U2OS cells were transiently transfected with GFP, GFP-Rab33b wt, GFP-Rab33b Q92L, or GFP-Rab33b T47N, lysed, and subjected to IP with GFP or control magnetic agarose beads. Whole-cell lysates (WCL) and immunoprecipitates (IP) were subjected to Western blot analysis using the indicated antibodies. (B) Coomassie blue staining of bacterially expressed His-Rab33b wt, His-Rab33b Q92L, His-Rab33b T47N, His-Rab9 wt, and His-Rab9 Q66L purified by using nickel-nitrilotriacetic acid (Ni-NTA) columns. (C) His-Rab33b wt, His-Rab33b Q92L, His-Rab33b T47N, His-Rab9 wt, and His-Rab9 Q66L were immobilized onto His-dynabeads® and incubated with cell lysate from U2OS cells to allow the binding of interactors. The samples were subjected to Western blot analysis using the indicated antibodies. (D) Representative image of a U2OS cell transfected with GFP-Rab33b wt and mCherry-Exoc6. Magnifications of the boxed areas show colocalization of Rab33b wt (green) and Exoc6 (magenta) on vesicles. Scale bar: 10 μm; insets: 1 μm. (E) The graph shows the normalized fluorescence intensity profile relative to Rab33b and Exoc6 along the lines as illustrated in the insets in (d), represented as mean ± SEM from 3 independent experiments. n = 119 vesicles from 15 cells. (F) The percentage of Exoc6 vesicles positive for Rab33b was calculated by using an object-based colocalization analysis with ImageJ software. The values represent the mean ± SEM from three independent experiments. n ≥ 30 cells. n.s., not significant (two-tailed paired Student′s t-test). See also Figure S3.

To shed more light into the mechanisms used by Rab33b to regulate cell migration, we looked for its interaction partners by a yeast two-hybrid screening. As Rab GTPases preferentially binds effector proteins when bound to GTP (Zhen and Stenmark, 2015), the constitutively active mutant Rab33b Q92L was used as bait to screen a human placenta cDNA library. Exoc6, a component of the exocyst complex, known as Sec15 in yeast (Martin-Urdiroz et al., 2016), was identified as a positive hit. Co-immunoprecipitation experiments performed in U2OS cells transiently transfected with either GFP-Rab33b wt, the constitutively active mutant GFP-Rab33b Q92L, or the dominant negative mutant GFP-Rab33b T47N confirmed the interaction (Figure 5A). Furthermore, we investigated whether the interaction was specific for Rab33b by pulldown experiments. For this, His-tagged Rab33b wt, Q92L, T47N, or His-tagged Rab9 wt or Q66L were expressed in bacteria, purified, and incubated with total extracts of U2OS cells (Figures 5B–5C). As shown in Figure 5C, only His-tagged Rab33b and not His-tagged Rab9 proteins pulled down Exoc6 from total cell extracts, thus demonstrating that the interaction is specific for Rab33b. Live imaging analysis further revealed that Rab33b and Exoc6 colocalize on circa 30% of the Exoc6-positive vesicles (Figures 5D–5F), suggesting that the interaction occurs on secretory vesicles. In line with the pulldown experiments, no significant difference in the percentage of colocalization was detected when GFP-Rab33b wt, GFP-Rab33b Q92L, or GFP-Rab33b T47N were expressed (Figures 5F and S3A–S3B). Moreover, both GFP-Rab33b wt and its constitutively active mutant were able to immunoprecipitate Exo70 and Sec10, two other subunits of the exocyst complex, indicating that Rab33b is involved in recruiting the complex (Figure S3C).

### Rab33b regulates Exoc6 localization at the leading edge of migrating cells by mediating a post-Golgi transport pathway to the plasma membrane

**Figure 6:**
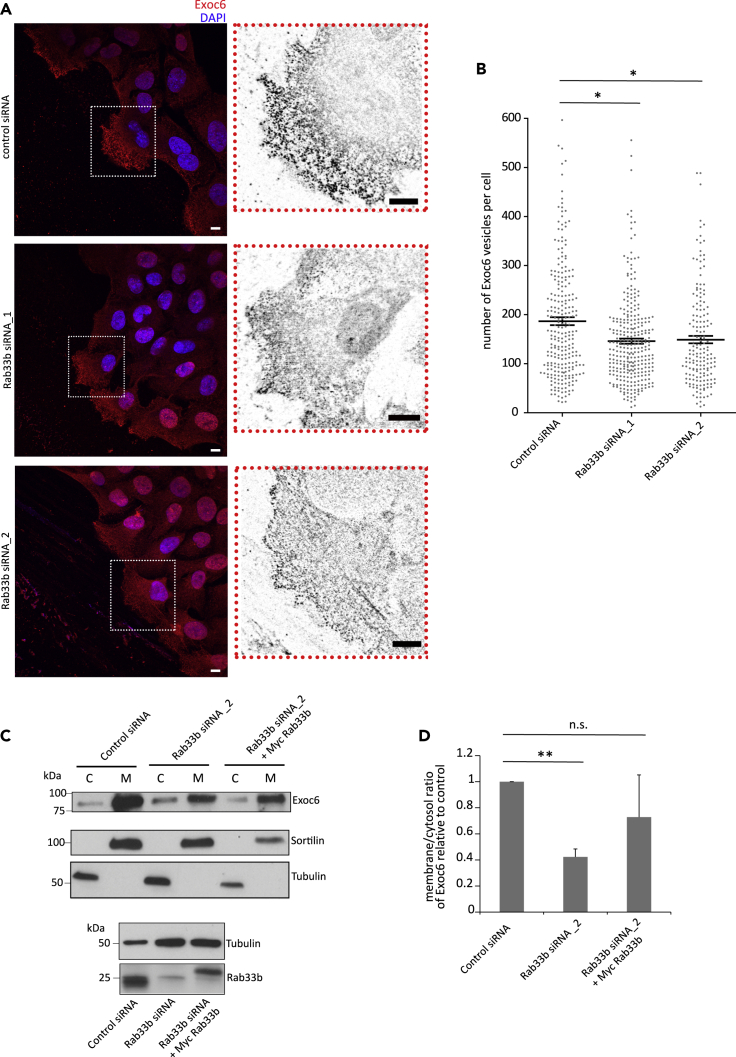
Membrane recruitment of Exoc6 is dependent on Rab33b (A) U2OS cells treated with siRNA control or Rab33b siRNA were grown to confluency, scratched with a pipet tip, and fixed after 6 h. Cells were immunostained with an antibody against Exoc6 and the nuclei labeled with DAPI. The insets show magnifications of the boxed areas. Scale bars: 10 μm. (B) Quantification of the number of Exoc6-positive vesicles per cell. The graph shows the mean ± SEM of a minimum of three independent experiments; n > 160 cells. ∗p < 0.05 (two-tailed paired Student′s t-test). (C) Upper panel: U2OS cells treated with siRNA control, Rab33b siRNA, or Rab33b siRNA and then transfected with Myc-Rab33b were subjected to ultracentrifugation to separate membrane (M) and cytosolic (C) fractions. The separated fractions were further analyzed by Western blot using an antibody against Exoc6. Antibodies against tubulin and sortilin were used as cytoplasmic and membrane-bound markers, respectively, to control separation between membrane and cytosolic fractions. Lower panel: Lysates from U2OS cells treated with siRNA control, Rab33b siRNA, or Rab33b siRNA and then transiently transfected with Myc-Rab33b were analyzed by Western blot using antibodies against Rab33b and tubulin as a loading control. (D) Quantification of the ratio between membrane-associated and cytosolic Exoc6 in U2OS cells treated with either Rab33b siRNA or Rab33b siRNA and then transiently transfected with Myc-Rab33b relative to the normalized siRNA control sample. The graph shows the mean ± SEM from four independent experiments. ∗∗p < 0.01, n.s., not significant (two-tailed paired Student′s t-test).

To characterize the role of the Rab33b-Exoc6 interaction, we next examined Exoc6 distribution upon Rab33b depletion by siRNA. Intriguingly, Rab33b knockdown reduced the number of Exoc6-positive vesicles at the leading edge of migrating cells of about 25%, suggesting that Exoc6 recruitment to secretory vesicles is dependent on Rab33b (Figures 6A–6B). In line with this, siRNA-mediated depletion of Rab33b decreased the fraction of Exoc6 associated to membranes, and the re-introduction of Rab33b was able to rescue this defect (Figures 6C–6D). Altogether, these results indicate that Rab33b is involved in the recruitment of Exoc6 to secretory vesicles.

**Figure 7:**
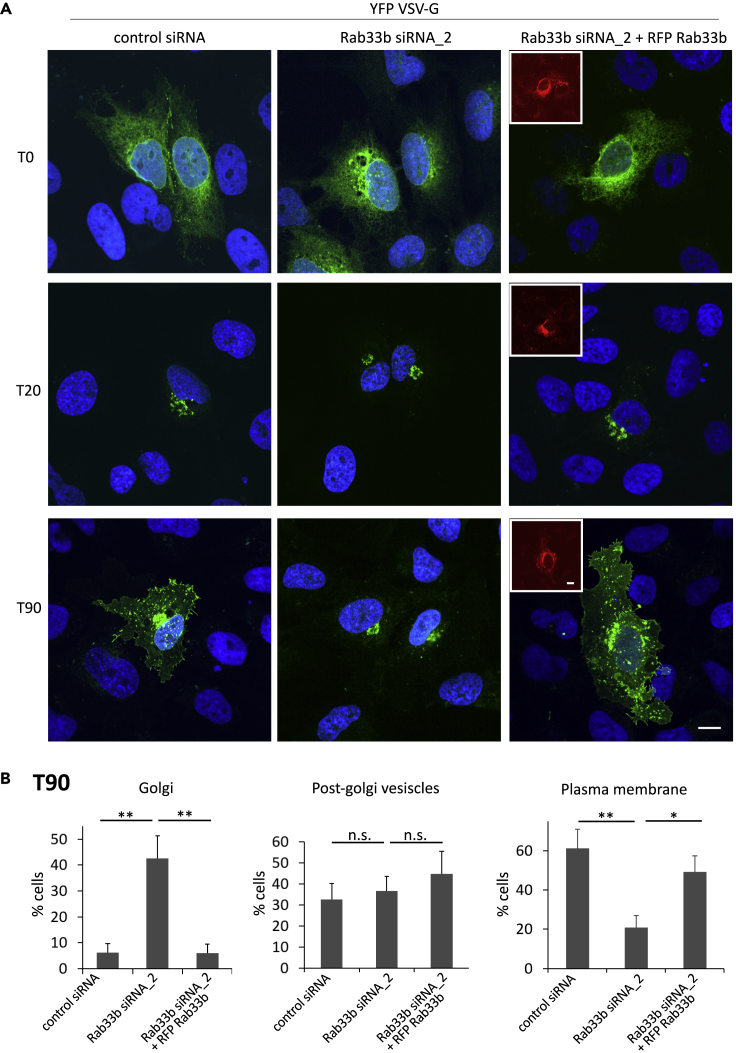
VSV-G transport to the cell surface is inhibited by Rab33b depletion (A) U2OS cells silenced with control siRNA, siRNA against Rab33b, or silenced with Rab33b siRNA and transfected with RFP-Rab33b (red), were transfected with YFP-VSV-G (green) and incubated at 39°C for 16 h. Cells were then fixed either immediately (T0), 20 min (T20), or 90 min (T90) after a shift to 32°C. Scale bar: 10 μm. (B) Quantification of the VSV-G distribution 90 min after the shift to 32°C. 200 cells were analyzed from three independent experiments and the percentage of cells in which YFP-VSV-G was located at the Golgi, post-Golgi vesicles, and plasma membrane was determined. The graph shows the mean ± SEM ∗p < 0.05, ∗∗p < 0.01, n.s., not significant (two-tailed paired Student′s t-test). See also Figure S4.

Rab33b has so far been reported to regulate Golgi-to-ER retrograde trafficking and Golgi homeostasis/organization (Starr et al., 2010; Valsdottir et al., 2001). Based on our findings about the interaction between Rab33b and Exoc6, and of the Rab33b-dependent localization of Exoc6 at the leading edge of migrating cells, we investigated whether Rab33b also regulates the secretory transport from the Golgi to the plasma membrane. We therefore analyzed vesicular stomatitis virus G protein (VSV-G) trafficking in cells depleted for Rab33b. YFP-VSV-G transfected cells were incubated at 39°C for 16 h to allow VSV-G accumulation in the ER. Then, the temperature was shifted to 32°C to synchronize the release of YFP-VSV-G from the ER into the secretory pathway. 20 min after the temperature shift to 32°C, YFP-VSV-G was present in the juxtanuclear Golgi apparatus in control cells, cells silenced for Rab33b or silenced and then transfected with RFP-Rab33b (Figure 7A). 90 min after the temperature shift, YFP-VSV-G was present on post-Golgi vesicles and at the plasma membrane in the vast majority of the control cells (>90%), as expected. Strikingly, YFP-VSV-G was unable to efficiently reach the plasma membrane in cells silenced for Rab33b, and it was still retained in the Golgi apparatus of over 40% of those cells. Re-introduction of Rab33b rescued the YFP-VSV-G transport to the plasma membrane similarly to control cells (Figures 7A–7B). A comparable defect in post-Golgi transport was detected in cells silenced for Rab33b by using the RUSH system to study the secretion of tumor necrosis factor-α (TNFα). TNFα-SBP-EGFP was retained in the ER by the Ii-streptavidin hook in both control and Rab33b-depleted cells. Addition of biotin released the transport of TNFα, which was detected in the Golgi apparatus after 12 min (Figure S4A). However, while TNFα had completely left the Golgi apparatus 90 min after biotin addition in the majority of control cells, over 60% of the cells silenced for Rab33b still presented TNFα localized at the Golgi (Figures S4A–S4B). These data confirm that Rab33b mediates a vesicular transport pathway from the Golgi apparatus to the plasma membrane.

### Rab33b regulates Exoc6-mediated secretion of cargos to FAs

**Figure 8:**
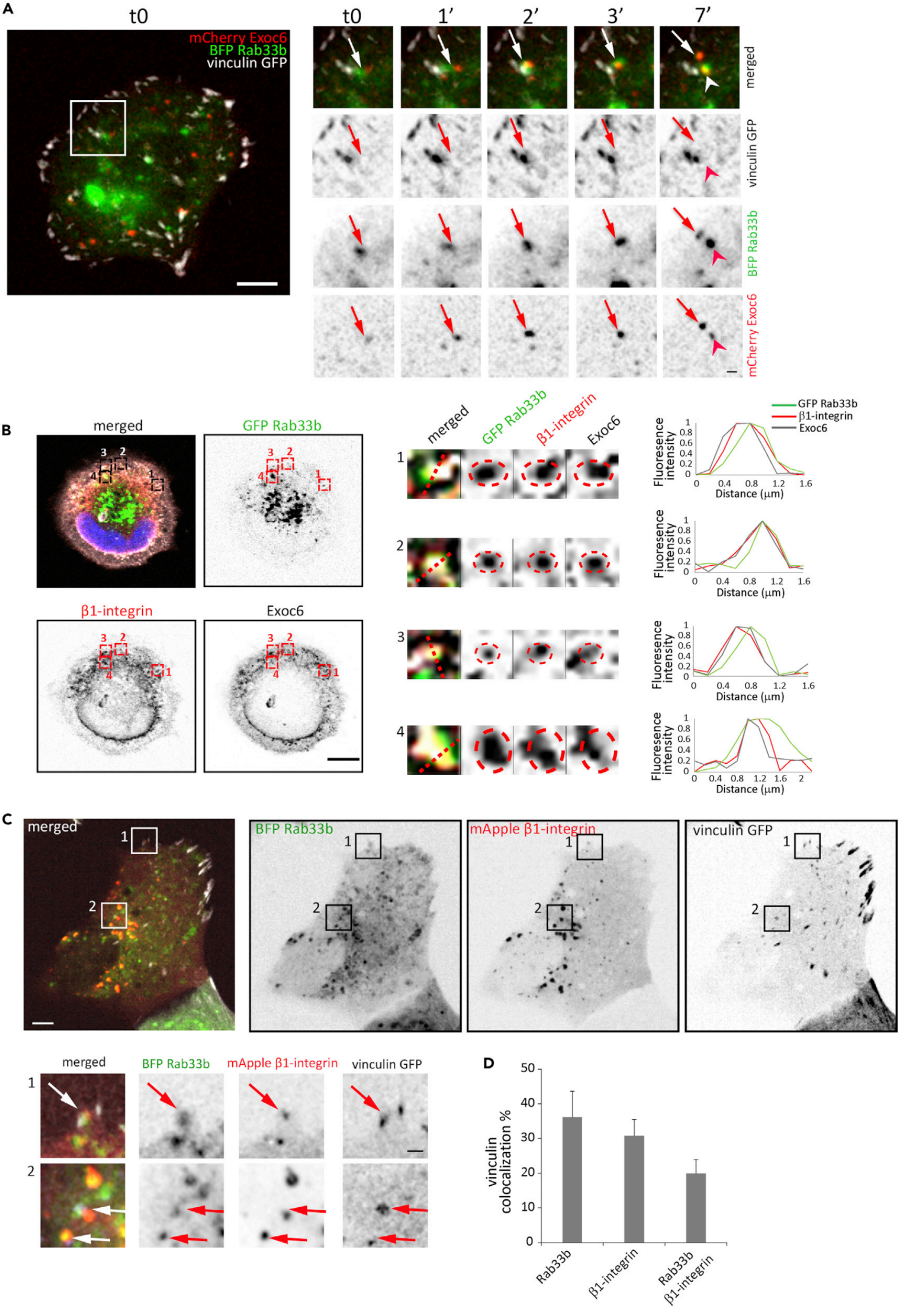
Rab33b and Exoc6 transport β1 integrin to FAs (A) U2OS cell transfected with BFP-Rab33b, mCherry-Exoc6, and vinculin-GFP were seeded on fibronectin-coated coverslips for 1 h. Cells were imaged every minute with a spinning disk confocal microscope. Magnifications of the boxed area in the right panels show Exoc6 together with Rab33b at FAs. Scale bars: 5 μm; insets: 1 μm. (B) U2OS cells transfected with GFP-Rab33b were seeded on fibronectin-coated coverslips for 1 h, fixed and then immunostained with antibodies against β1-integrin and Exoc6. DAPI was used to stain the nuclei. The insets show magnifications of the boxed areas. Red circles are drawn around Rab33b-positive vesicles and superimposed in the β1-integrin and Exoc6 channels. Normalized fluorescence intensity profiles along the straight red lines are shown on the right for each channel. Scale bar: 10 μm. (C) U2OS cells transfected with BFP-Rab33b, mApple-β1 integrin, and vinculin-GFP were seeded on fibronectin-coated coverslips for 1 h. Cells were imaged with a spinning disk confocal microscope. The insets show Rab33b (green) and β1 integrin (red) co-localizing on vesicles in proximity of FAs (white). Scale bars: 5 μm; insets: 1 μm. (D) The percentage of vinculin-GFP-positive focal adhesions co-localizing with BFP-Rab33b, mApple-β1 integrin, or both, was calculated by using an object-based colocalization analysis with ImageJ software. The graph shows the mean ± SEM from three independent experiments. n = 9. See also Video S4.

The exocyst complex mediates the targeted tethering of secretory vesicles to sites of exocytosis. These post-Golgi vesicles deliver biosynthetic cargo, including integrins, to the leading edge of migrating cells (Spiczka and Yeaman, 2008). We therefore hypothesize that Rab33b may regulate focal adhesion turnover by mediating the transport and targeted delivery of adhesion molecules at cell protrusions together with Exoc6. To test this hypothesis, we first evaluated whether Rab33b is present together with Exoc6 in proximity of FAs. For this, we followed the live dynamics of Rab33b and Exoc6 toward forming FAs in cells spreading on fibronectin-coated coverslips. Microscopy analysis revealed Exoc6-positive vesicles contacting Rab33b-positive vesicles at FAs (Figure 8A and Video S4). Moreover, immunofluorescence analysis showed that these Rab33b- and Exoc6-positive vesicles contained β1-integrin (Figure 8B), and live-cell imaging further confirmed that the Rab33b-positive vesicles in proximity of FAs contain β1-integrin (Figures 8C–8D). Overall, this suggests that Rab33b, together with Exoc6, delivers integrin to adhesion sites to regulate FA turnover, revealing the mechanism used by Rab33b to regulate cell migration.

Video S3. Rab33b-positive vesicles are delivered to growing FAs during membrane protrusion,U2OS cells transiently transfected with GFP-Rab33b (green) and vinculin-RFP (red) were imaged every 30 s using a TIRF microscope with a penetration depth of 90 nm. The movie shows the recruitment of GFP-Rab33b-positive vesicles during membrane protrusion. Related to Figure 4.

## Discussion

In addition to their established role in regulating intracellular membrane transport, Rab GTPases can regulate several other cellular processes. In the last decade, various works have shown the role of different members of the Rab family in cell migration (Borg et al., 2014; Caswell et al., 2007; Palamidessi et al., 2008; Vestre et al., 2019). Here, we performed an siRNA screen to reveal previously uncharacterized Rab proteins involved in cell migration and identified Rab33b as a negative regulator of cell motility. Rab33b has so far been described to localize at the Golgi apparatus where it regulates Golgi-to-ER retrograde trafficking and Golgi homeostasis/organization (Starr et al., 2010; Valsdottir et al., 2001), and to modulate the formation of autophagosomes (Itoh et al., 2008; Pantoom et al., 2021). However, based on the current limited knowledge about Rab33b function, it was difficult to understand how this small GTPase could regulate cell migration. We therefore performed a yeast two-hybrid screening to find potential interaction partners of Rab33b, and identified the exocyst subunit Exoc6 as a putative Rab33b interactor. Interestingly, we confirmed by co-immunoprecipitation and pulldown experiments that Rab33b interacts with Exoc6 independently of its nucleotide state. Even though Rab proteins mostly interact with their effectors when they are in a GTP-bound form, there are also interactors that bind to Rabs in a nucleotide-independent manner (Burke et al., 2014; Lindsay and McCaffrey, 2002; Miserey-Lenkei et al., 2007).

Intriguingly, another interactor of Rab33b, ATG16L1, similarly to Exoc6 also binds weakly to the Rab33b dominant negative mutant (Metje-Sprink et al., 2020). Recently, it has been reported that this is possible because ATG16L1 can induce Rab33b to adopt an active conformation without nucleotide exchange (Pantoom et al., 2021). In line with the ability of Rab33b dominant negative mutant to recruit interactors, its intracellular localization is not cytosolic as for the majority of the Rabs when bound to GDP, but membrane-associated (Figure S3B and (Metje-Sprink et al., 2020)). Furthermore, Exoc6 has a percentage of colocalization with Rab33b T47N only slightly decreased compared to the wt or constitutively active mutant of Rab33b. This is in agreement with the pulldown assays that show a weaker, but still detectable interaction between Rab33b T47N and Exoc6. Noteworthy, the expression of Rab33b dominant negative mutant does not influence cell migration. Altogether, this suggests that Rab33b T47N does not behave like a loss-of-function mutant.

Exoc6 is one of the subunits of the exocyst complex. This complex is required for polarized exocytosis and mediates the tethering of post-Golgi secretory vesicles to the plasma membrane before exocytic fusion (He and Guo, 2009; Mei and Guo, 2018). The exocyst complex is also important for cell migration, as it is implicated in the delivery of proteins, including adhesion molecules and proteases for extracellular matrix (ECM) degradation, to the leading edge of migrating cells (Liu and Guo, 2012). However, the exact mechanism used by the exocyst complex to regulate cell migration is still poorly characterized. The depletion of some subunits such as Sec5 and Sec6 reduces cell migration (Spiczka and Yeaman, 2008), while silencing of other subunits, like Sec10, increases cell migration (Noh et al., 2018). This suggests that the different subunits of the exocyst complex might also work differently from each other. In line with this, structural studies of the complex have identified that the eight components of the exocyst are divided into two subcomplexes, where subcomplex I consists of Sec3-Sec5-Sec6-Sec8 while subcomplex II consists of Sec10-Sec15-Exo70-Exo84 (Mei and Guo, 2018). The two subcomplexes can assemble, associate with vesicles, and localize to the PM independently of each other (Ahmed et al., 2018). Moreover, Sec5 and Sec6 (subcomplex I) are highly enriched in the pseudopods, while Sec10 and Sec15/Exoc6 (subcomplex II) are not (Spiczka and Yeaman, 2008). It is therefore possible that the two exocyst subcomplexes may differently regulate cellular functions/pathways. In agreement with this hypothesis, Sec5 and Sec6, the subunits that when knocked down decrease cell migration, are part of subcomplex I, while Sec10 (its depletion increases cell migration) and Exoc6/Sec15 are both in subcomplex II.

Therefore, the role of Rab33b in cell migration is likely linked to its interaction with the exocyst component Exoc6. This is indeed in line with our results showing that the depletion of Rab33b prevents Exoc6 recruitment to membranes and decreases the number of Exoc6 vesicles present at the leading edge of migrating cells, and that Exoc6 localizes to Rab33b-positive vesicles. We further demonstrated for the first time that Rab33b has a role in the transport of secretory cargo from the Golgi to the plasma membrane. Altogether, these data support the role of Rab33b in the recruitment and association of Exoc6 to Rab33b-positive secretory vesicles.

Even though a direct involvement of Rab33b in the regulation of the secretory pathway has never been reported before, previous published evidence is in line with this newly identified function. For example, Rab33b has recently been shown to mediate axon outgrowth during the development of zebrafish brain (Huang et al., 2019). Although the underlying molecular mechanisms are still unknown, it has been hypothesized that this occurs through the regulation of the anterograde axonal transport of post-Golgi vesicles and their exocytosis at the growth cone. Furthermore, the effect of Rab33b silencing seems to phenocopy the effect of Rab6 silencing, a small GTPase that regulates the transport of post-Golgi vesicles (Fourriere et al., 2019; Grigoriev et al., 2007; Miserey-Lenkei et al., 2017). Indeed, similarly to Rab33b depletion, Rab6 depletion promotes cell migration (Vestre et al., 2019) and delays protein secretion (Fourriere et al., 2019). It is therefore tempting to speculate that Rab6 and Rab33b involvement in cell migration might be linked to their function in the secretory pathway. Interestingly, it has been suggested that Rab33b and Rab6 are part of a Rab cascade at the Golgi (Pusapati et al., 2012), and it is likely that a similar Rab cascade may occur also on secretory vesicles. As Rab6 interacts with several kinesin motors (Echard et al., 1998; Fourriere et al., 2019; Lee et al., 2015; Miserey-Lenkei et al., 2017), if the two Rabs are involved in a Rab cascade, it is likely that also Rab33b will bind to a kinesin to mediate the movement of secretory vesicles toward the plasma membrane. The delay in secretion upon Rab33b silencing may therefore be a consequence of the defective transport of secretory vesicles along microtubules due to the lack of interaction between Rab33b and the molecular motor.

Migrating cells form integrin-mediated contacts with the ECM, thus connecting the ECM to the actin cytoskeleton. These forming adhesion points mature to become larger and more stable FAs that serve as anchorage points for the cell. A tight coordination of assembly/disassembly of FAs is fundamental for proper cell migration. Our results demonstrate that Rab33b-positive vesicles are directed to FAs, and that silencing of Rab33b promotes FAs disassembly, thus indicating that Rab33b recruitment to FAs is required for their stability. This is further supported by our live-cell imaging studies revealing that Rab33b-positive vesicles accumulate at areas of cell protrusions where new adhesions are formed and need to be stabilized. The increased FA disassembly upon Rab33b depletion will then result in a greater capacity of the cell to move forward. This is in line with the knowledge that faster FA disassembly at plasma membrane protrusions is a feature of invasive compared with non-invasive cancer cells and it is also critical for cells to become motile, while cells with more stable FAs are generally less motile (Bijian et al., 2013; Xu et al., 2010). Intriguingly, analysis of The Cancer Genome Atlas (TCGA) dataset shows that Rab33b expression is downregulated in lung cancer compared to normal tissues (Figure S5A). Moreover, analysis of Rab33b expression using the TNM plot analysis revealed that the expression further decreases with the progression of the disease and metastasis (Figure S5B). Rab33b expression also correlates with Exoc6 expression (Figure S5C), suggesting a relevance for the Rab33b-Exoc6 axis in cancer.

The exocyst complex interacts with paxillin, a component of FAs, at protrusions, possibly contributing to the turnover of cell adhesions to substrate (Spiczka and Yeaman, 2008). Therefore, it is tempting to speculate that Rab33b-positive secretory vesicles contribute to the recruitment of Exoc6 in proximity of FAs to mediate the localized delivery of adhesion components for the correct turnover of FAs. In agreement with this, we observed both the recruitment of Exoc6 to Rab33b-positive vesicles at FAs in control cells, and impaired localization of Exoc6 at the leading edge of migrating cells upon Rab33b knockdown.

Which cargo is transported along this pathway? It is well established that the exocyst contributes to integrin trafficking to the leading edge of migrating cells (Polgar and Fogelgren, 2018; Spiczka and Yeaman, 2008). Accordingly, we could detect β1-integrin together with Rab33b and Exoc6 at FAs, suggesting that the interaction between Rab33b and Exoc6 is important for the delivery of β1-integrin to support FAs stability.

In sum, our work reveals that Rab33b, by interacting with Exoc6, regulates the localized delivery of post-Golgi vesicles transporting cargo such as integrins to FA sites, thereby influencing the dynamics of FAs that are required for proper cellular migration.

### Limitations of the study

The present work elucidates the role of Rab33b in cell migration *in vitro.* Cell migration is a very complex process, which is also influenced by multiple extracellular factors that cannot be reproduced *in vitro*. Therefore, further work is required to elucidate the physiological consequences of the observed *in vitro* effects with *in vivo* studies. Moreover, despite our analysis of The Cancer Genome Atlas (TCGA) datasets indicates a correlation between Rab33b and Exoc6 expression in cancer, the precise role of Rab33b-Exoc6 axis in cancer progression and metastasis has not been investigated and will require future studies.

## STAR★Methods

### Key resources table

**Table:** 

REAGENT or RESOURCE	SOURCE	IDENTIFIER
**Antibodies**		

GFP-Trap® agarose beads	ChromTek	Cat# gta-20; RRID:AB_2631357
Rabbit polyclonal anti-giantin	Abcam	Cat# ab24586; RRID:AB_448163
Mouse monoclonal anti-vinculin	Sigma-Aldrich	Cat# V9131; RRID:AB_477629
Rabbit polyclonal anti-GFP	Abcam	Cat# ab6556; RRID:AB_305564
Mouse monoclonal anti-tubulin	Life Technologies	Cat# 13-8000; RRID:AB_2533035
Rabbit polyclonal anti-exoc6	Sigma-Aldrich	Cat# HPA036285; RRID:AB_10673617
Rabbit polyclonal anti-exoc6	Abcam	Abcam Cat# ab104200; RRID:AB_10716450
Mouse monoclonal anti-β_1_ integrin	Santa Cruz	Cat# sc-59829; RRID:AB_782090
Rabbit polyclonal anti-sortilin	Abcam	Cat# ab16640; RRID:AB_2192606
Monoclonal mouse anti-Rab33b	Abcam	Cat# ab57794; RRID:AB_945116
Monoclonal mouse anti-histidine	AbD serotec	Cat# MCA1396; RRID:AB_322084
Monoclonal mouse anti-GM130	BD Transduction Laboratories	Cat# 610822; RRID:AB_398141
Monoclonal mouse anti-Exo70	Santa Cruz	Cat# sc-365825; RRID:AB_10843358
Mouse monoclonal anti-Sec10	Santa Cruz	Cat# sc-514802; RRID:AB_2891091
Goat anti-Mouse IgG(H + L) Cross-Adsorbed Secondary Antibody, Alexa Fluor 488	Invitrogen	Cat# A-21121; RRID:AB_2535764
Goat anti-Rabbit IgG(H + L) Cross-Adsorbed Secondary Antibody, Alexa Fluor 488	Invitrogen	Cat# A27034; RRID:AB_2610664
Goat anti-Mouse IgG(H + L) Cross-Adsorbed Secondary Antibody, Alexa Fluor 594	Invitrogen	Cat# A-21155; RRID:AB_2535785
Goat anti-Rabbit IgG(H + L) Cross-Adsorbed Secondary Antibody, Alexa Fluor 594	Invitrogen	Cat# A-11012; RRID:AB_2534079
Goat anti-Mouse IgG(H + L) Cross-Adsorbed Secondary Antibody, Alexa Fluor 647	Invitrogen	Cat# A-21240; RRID:AB_2535809
Goat anti-Rabbit IgG(H + L) Cross-Adsorbed Secondary Antibody, Alexa Fluor 647	Invitrogen	Cat# A-21244; RRID:AB_2535812

**Bacterial and virus strains**		

*Escherichia coli* BL21 (DE3)	Agilent Technologies	Cat# 200131

**Chemicals, peptides, and recombinant proteins**		

DAPI ((2-(4-Amidinophenyl)-6-indolecarbamidine)	Sigma-Aldrich	Cat# D9542
Rhodamine-conjugated phalloidin	Invitrogen	Cat# R415
Lipofectamin 2000	Life Technologies	Cat# 10696153
Lipofectamin RNAiMax	Life Technologies	Cat# 13778150
His-dynabeads®	Thermo Fisher	Cat# 10103D
D-biotin	Sigma-Aldrich	Cat# B4501
Fibronectin	Sigma	Cat# F2006

**Critical commercial assays**		

Yeast two-hybrid screen	Hybrigenics services	N/A

**Experimental models: Cell lines**		

U-2 OS	ATCC	Cat# HTB-96; RRID:CVCL_0042
hTERT RPE-1	ATCC	Cat# CRL-4000; RRID:CVCL_4388

**Oligonucleotides**		

siRNA targeting sequence: Rab33b (siRNA_1)	Dharmacon	Cat# J-008909-07
siRNA targeting sequence: Rab33b (siRNA_2)	Dharmacon	Cat# J-008909-08
siRNA targeting sequence: Rab33b (siRNA_3)	Dharmacon	Cat# J-008909-06
siRNA targeting sequence: Rab33b (siRNA_4)	Dharmacon	Cat# J-008909-09
Control siRNA: ACU UCG AGC GUG CAU GGC UTT	MWG-Biotech	N/A
siRNAs used in the RNAi primary screen, see Table S1	Dharmacon	N/A

**Recombinant DNA**		

pEGFP-C1 vector	BD Bioscience Clontech	Cat# 6084-1
pEGFP-C1 Rab33b wt	GenScript	N/A
pEGFP-C1 Rab33b Q92L	GenScript	N/A
pEGFP-C1 Rab33b T47N	GenScript	N/A
pcDNA3.1-N-myc Rab33b wt	GenScript	N/A
pET-16b Rab33b wt	GenScript	N/A
pET-16b Rab33b Q92L	GenScript	N/A
pET-16b Rab33b T47N	GenScript	N/A
pET-16b Rab9 wt	Progida et al., 2012	N/A
pET-16b Rab9 Q66L	This paper	N/A
pCAG-mCherry-C-Sec15A	Kazuhisa Nakayama. Katoh et al., 2015	N/A
RFP-vinculin	Harald Stenmark. Lobert and Stenmark, 2012	N/A
Vinculin-GFP	Xian Hu	N/A
mApple-Integrin-β1	Addgene (from Michael Davidson)	Addgene plasmid # 54914; RRID:Addgene_54914
YFP-VSV-G	Susanne Pfeffer. Espinosa et al., 2009	N/A
BFP-Rab33b	This paper	N/A
Ii-STR-TNF-SBP-EGFP	Addgene (from Franck Perez). Boncompain et al., 2012	Addgene plasmid # 65280; RRID:Addgene_65280

**Software and algorithms**		

ImageJ	https://imagej.nih.gov/ij/	N/A
GraphPad Prism 9	GraphPad Software Inc., https://www.graphpad.com	N/A
IncuCyte Zoom software analysis	Essen bioscience	N/A

### Resource availability

#### Lead contact

Further information and requests for resources and reagents should be directed to and will be fulfilled by the lead contact, Cinzia Progida (c.a.m.progida@ibv.uio.no).

#### Materials availability

All unique reagents generated in this study are available from the lead contact upon request.

### Experimental model and subject details

#### Cell lines

U2OS cells were grown in Dulbeccós modified Eaglés medium (DMEM; Lonza, BioWhittaker). RPE-1 cells were grown DMEM F-12 (Lonza, BioWhittaker). Both DMEM and DMEM F-12 were supplemented with 10% fetal calf serum (FCS), 2 mM L-glutamine, 100 U/mL penicillin, and 100 μg/mL streptomycin. Cells were maintained in a 5% CO_2_ atmosphere at 37°C.

### Method details

#### Constructs and antibodies

pEGFP-C1 Rab33b wt, pEGFP-C1 Rab33b Q92L, pEGFP-C1 Rab33b T47N, pcDNA3.1-N-myc Rab33b wt, pET-16b Rab33b wt, pET-16b Rab33b Q92L, and pET-16b Rab33b T47N were purchased from GenScript. pET-16b Rab9 wt has been described previously (Progida et al., 2012). pET-16b Rab9 Q66L was obtained by amplifying Rab9 Q66L by PCR using the following primers: 5′-AGAGAGATATCATATGGCAGGAAAATCTTCACTT-3′, and 5′-AGAGACTCGAGTCAACAGCAAGATGAGCTAGG-3′. The fragments were cut with *Nde*I and XhoI and then inserted into pET16b, cut with the same restriction enzymes, in frame with poly-His. The construct was sequenced to exclude the presence of Taq polymerase mistakes. pEGFP-C1 was purchased from BD Biosciences Clontech. pCAG-mCherry-C-Sec15A was kindly provided by Prof. Kazuhisa Nakayama (Kyoto University, Kyoto, Japan) (Katoh et al., 2015). RFP-vinculin was a kind gift from Harald Stenmark (Institute for Cancer Research, Oslo University Hospital, Oslo, Norway). Vinculin-GFP was a gift from Xian Hu (Centre for Molecular Medicine Norway, University of Oslo, Oslo, Norway). mApple-Integrin-β1 was a gift from Michael Davidson (Addgene plasmid # 54914; http://n2t.net/addgene:54914; RRID:Addgene_54914). YFP-VSV-G plasmid was a kind gift of Susanne Pfeffer (Stanford University, CA) (Espinosa et al., 2009). BFP-Rab33b was generated by cloning Rab33b wt gene in the EBFP2-C1 vector by using BspEI and BamHI restriction enzymes. The primary antibodies used in this study for western blot (WB) and immunofluorescence (IF) analysis include anti-giantin (Abcam, ab24586, IF 1:1,000), anti-vinculin (Sigma Aldrich, IF 1:150), anti-GFP (Abcam, ab6556, WB 1:1000), anti-tubulin (Life Technologies, #13-8000, WB 1:24000), anti-exoc6 (Sigma Aldrich, HPA036285, WB 1:300), anti-exoc6 (Abcam, ab104200, IF 1:300), anti-β_1_ integrin (Santa Cruz, sc-59829, IF 1:100), anti-sortilin (Abcam, ab16640, WB 1:000), anti-Rab33b (Abcam, ab57794, WB 1:200), anti-histidine (AbDserotec, MCA1396, WB 1:1000), anti-GM130 (BD Transduction Laboratories, 610822, IF 1:300), anti-Exo70 (Santa Cruz, sc-365825, WB 1:100), anti-Sec10 (Santa Cruz, sc-514802, WB 1:100). For nuclear staining, DAPI (Sigma-Aldrich) was used at 0.1 μg/mL. Rhodamine-conjugated phalloidin was purchased from Invitrogen (R415) and used at 1:200. Alexa Fluor secondary antibodies (Invitrogen) were used for immunofluorescence at dilution 1:200 and secondary antibodies conjugated to horseradish peroxidase for immunoblotting studies (GE Healthcare) were diluted 1:5000.

#### Transfection and RNA interference

For transient transfection of U2OS cells Lipofectamine 2000 (Life Technologies) was used according to the manufactureŕs protocol. The cells were transfected 24 h prior to further execution of experiments at a confluency of approximately 50%–70%.

Transfection with siRNAs was done using Lipofectamine RNAiMAX Transfection Reagent (Life Technologies) following the produceŕs protocol. The cells were transfected 72 h prior to analysis. Reverse transfection was used for all assays performed in 96 well plates, including all migration and proliferation assays performed using the IncuCyte system, and the micropattern experiments. The siRNA pools used in the screens (Dharmacon) are listed in Table S1. The single siRNAs targeting Rab33b were purchased from Dharmacon (Rab33b siRNA_1: J-008909-07; Rab33b siRNA_2: J-008909-08; Rab33b siRNA_3: J-008909-06; Rab33b siRNA_4: J-008909-09). Non-targeting control siRNA (sense sequence 5′-ACUUCGAGCGUGCAUGGCUTT-3′ and antisense 5′-AGCCAUGCACGCUCGAAGUTT-3′) was purchased from MWG-Biotech (Ebersberg, Germany).

#### Yeast two-hybrid screen

A yeast two-hybrid screen of a human cDNA library from placenta using human Rab33bΔC Q92L as bait was performed by Hybrigenics services (Paris, France).

#### Co-immunoprecipitation

The GFP-Trap®_MA (Chromotek) was used for co-immuniprecipitation experiments according to the produceŕs protocol. Briefly, cells transiently transfected with GFP-fusion proteins were lysed in lysis buffer (10 mM Tris-HCl pH 7.5, 150 mM NaCl, 0.5 mM EDTA, and 0.1% NP-40) and the lysates incubated for 1 h at 4°C with magnetic beads coupled to anti-GFP or non-coupled magnetic beads as control. The immunoprecipitated samples and their respective total lysates were loaded on SDS-PAGE gels and analyzed by western blotting.

#### Protein purification and pulldown experiments

His-tagged Rab proteins were expressed in *Escherichia coli* BL21 (DE3) (Agilent Technologies) after induction with 0.5 mM IPTG for 4 h at 37°C. The bacterial cultures were centrifuged and the pellets were resuspended in buffer containing 64 mM Tris-HCl pH 8.5, 8mM MgCl_2_, 20 mM β-mercaptoethanol, 0.30 mM PMSF, 0.8 ng lysozyme/gram pellet and 10 μg/mL DNase. The resuspended bacterial cultures were lysed by French press and centrifuged at 48,000 × g for 1 h at 4°C. The soluble fractions containing the expressed His-tagged Rab proteins were purified by using Nickel-nitrilotriacetic acid (Ni-NTA) columns. Amicon® Ultra-15 filtration tubes were used to concentrate the purified proteins and the buffer exchanged with PBS 1×. Pulldown experiments were performed by using magnetic His-dynabeads® (Thermo Fisher). 12 μg of purified His-tagged proteins were bound to Dynabeads and incubated with 200 μL of precleared U2OS cell lysates for 30 min at 4°C. To activate Rab GTPases, purified His-tagged Rabs bound to Dynabeads were loaded with 0.1 mM GTPγS. The beads were washed ten times with buffer containing 3.25 mM Na-P pH 7.4, 79 mM NaCl, 0.01% Tween 20. Bound proteins were eluted with elution buffer (50 mM Na-P pH 8.0, 300 mM NaCl, 0.01% Tween 20, 300 mM Imidazole). Samples were analyzed by using SDS-PAGE and immunoblotting.

#### Subcellular fractionation assay

U2OS cells were washed with 1× PBS, scraped and collected before centrifugation at 1300 × g for 5 min at 4°C. To lyse the cells, 250 μL homogenizer buffer (8% sucrose, 3 mM imidazole and 1:100 protease inhibitor) was added to each sample. A 27 G syringe was used to mechanically lyse the cells, before centrifugation at 3800g for 15 min at 4°C to pellet the nuclei. The supernatant was further centrifuged at 100,000 × g for 1 h at 4°C, using a TLA 100.1 rotor (Beckman Coulter). After ultracentrifugation, supernatant (i.e. cytosol) and pellet (i.e. membranes) were carefully separated and subjected to western blot analysis. ImageJ was used to quantify the levels of Exoc6 signal from the membrane and cytosolic fractions. For each condition, the ratio between the levels of Exoc6 in the membrane and cytosolic fractions was calculated.

#### Immunoblotting

Cells were lysed in lysis buffer (125 mM K-acetate, 25 mM Hepes, 5 mM EGTA, and 2.5 mM Mg-acetate, pH 7.2) complemented with 0.5% NP-40, protease inhibitor cocktail, and DTT. Cell lysates were subjected to SDS-PAGE followed by blotting onto polyvinylidene fluoride (PVDF) membranes (Millipore) and incubation with primary antibodies diluted in 2% blotting grade non-fat dry milk (BioRad). Next, the membranes were incubated with secondary antibodies conjugated to horseradish peroxidase (HRP) (GE Healthcare). For chemiluminescence detection, either the ECL Prime Western Blotting Detection (GE Healthcare) or SuperSignal West Femto Maximum Sensitivity Substrate (Thermo Scientific) were used followed by imaging using a Kodak Image Station 4000R equipped with a CCD camera. Densitometry analysis of band intensity was done using the Carestream software.

#### Wound healing assays

U2OS cells were reversely transfected in IncuCyte ImageLock 96-well plates (Essen Bioscience) and grown into confluent monolayers before wounding with the IncuCyte WoundMaker (Essen Bioscience). Imaging was performed by using the IncuCyte ZOOM with a 10× objective (Essen Bioscience), and the relative wound density calculated by the IncuCyte ZOOM software analysis program. For individual cell tracking and analysis of cell speed, the Fiji/ImageJ manual tracking plugin and Ibidi Chemotaxis software was used.

#### Cell proliferation assay

Cells were reversely transfected in a 96-well plate and subsequently imaged for 72 h with an IncuCyte ZOOM using a 10× objective. Cell confluency was measured using the IncuCyte ZOOM software analysis program.

#### VSV-G secretion assay

Cells grown on coverslips were transfected with YFP-VSV-G plasmid. At 2 h after transfection, cells were shifted to 39°C for 16 h. To release VSV-G, cells were transferred to 32°C and the samples were fixed at the indicated times.

#### RUSH assay

U2OS cells grown on coverslips were transiently transfected with Ii-STR-TNF-SBP-EGFP (a gift from Franck Perez, Addgene plasmid # 65280; http://n2t.net/addgene:65280; RRID:Addgene_65280 (Boncompain et al., 2012)) overnight. The day after, the cells were incubated with 40 μM D-biotin (Sigma-Aldrich) for 12 min, 60 min or 90 min at 37 °C, then fixed with 3% paraformaldehyde and processed for immunofluorescence microscopy.

#### Golgi re-oritentation measurement

Cells seeded on cover slips were grown into confluent monolayers and scratched with a pipette tip, followed by a 5 h incubation at 37°C and 5% CO_2_ before fixation and staining with anti-giantin, rhodamine-conjugated phalloidin and DAPI to visualize the Golgi complex, actin cytoskeleton and nuclei, respectively. For the snalysis of Golgi re-orientation, the cells at the edge of the wound were divided into three equal sectors. The percentage of cells having their Golgi apparatus in the front sector between the nucleus and the leading edge was calculated.

#### Micropatterns

96-well plates (CYTOO, 20-900-00) containing circular or crossbow-shaped micropatterns (1100 μm^2^) were coated with 20 μg/mL sterile fibronectin (Sigma F2006) for 30 min. The wells were then washed with PBS 1× and 3 × 10^3^ cells were seeded in each well. Cells were kept for 3.5 h at 37°C and 5% CO_2_, before fixation and staining with rhodamine-conjugated phalloidin and an anti-vinculin or anti-giantin antibodies. The CYTOOL-IP ReferenceCell macro in ImageJ was used to analyze the actin, vinculin, and giantin distribution of cells plated on micropatterns. This macro allows the alignment and averaging of several micropatterned cells, generating a color-coded frequency map for a normalized mean cell that provides a statistical representation of the actin, vinculin, and giantin intracellular distribution.

#### Immunofluorescence microscopy

Cells seeded on cover slips were fixed using 3% paraformaldehyde, quenched with 50 mM NH_4_Cl, and permeabilized with 0.25% saponin in 1× PBS. Both primary and secondary antibodies were added to the cells at room temperature for 20 min before mounting with Mowiol. Imaging acquisition was done using an Olympus FluoView 1000 IX81 confocal laser scanning microscope (inverted) with a 60× PlanApo NA 1.35 objective.

#### Live cell imaging

For live-cell imaging, cells were seeded on MatTek glass-bottom dishes. During imaging, the cells were kept at 37 °C and 5% CO2. For studies of FA dynamics, multiple positions were imaged every 20 min using an inverted Olympus FluoView 1000 IX81 confocal laser scanning microscope equipped with a 60× PlanApo NA 1.35 objective. For imaging of the Rab33b-vinculin contact points, a Zeiss LSM880 microscope equipped with a 63× oil Plan Apo NA 1 objective was used. Total internal reflection fluorescence (TIRF) microscopy acquisition was done by using a TIRF Leica microscope using a 100× objective. Images were taken every 30 s with 90 nm penetration depth. For all the other live-cell studies, cells were imaged by using the spinning disk Andor Dragonfly with a 60× Apo objective, NA 1.4.

#### Image processing and analysis

Image analysis and processing was performed using ImageJ (National Institutes of Health) and Adobe Photoshop (Adobe Systems).

#### Dataset analysis

RNA expression datasets and clinical information were downloaded from the TCGA (https://portal.gdc.cancer.gov/), (Cerami et al., 2012; Gao et al., 2013) with the use of the FireBrowse Data Portal (http://firebrowse.org/) (Deng et al., 2017). The expression levels of Rab33b mRNA (RSEM log2) were assessed in lung cancer tissues relative to its expression in normal tissues. The statistical significance was determined by Student′s two-tailed homoscedastic t-test. Comparison of Rab33b expression in normal, primary tumor and metastatic tissues was obtained from gene chip data at TNMplot.com (Bartha and Gyorffy, 2021). For the correlation analysis, lung adenocarcinoma gene expression profile dataset available at the cBioCancer Genomics Portal (TCGA, PanCancer Atlas) was used. The samples (n = 566) were aligned by the expression level of Rab33b. The subgroups showing the highest (n = 30) and the lowest (n = 30) Rab33b expression were then used for possible correlation with the expression of Exoc6. The correlation test was further applied to another lung adenocarcinoma gene expression profile dataset (Chen et al., 2020), (n = 305), using the same methodology. The results are displayed in a heatmap generated in Excel. The correlation values were determined by the correlation function in Excel.

### Quantification and statistical analysis

Statistical analysis was done using the two-tailed paired Student′s t-test in Excel (Microsoft). In the figures, statistical significance is indicated as follows: ∗p < 0.05, ∗∗p < 0.01, ∗∗∗p < 0.001. The statistical details of experiments are given in the figure legends. Results are expressed as means ± SEM.

## Data Availability

All data reported in this paper will be shared by the lead contact upon request. This paper does not report original code. Any additional information required to reanalyze the data reported in this paper is available from the lead contact upon request.

## Supplemental information

Video S4. Exoc6- and Rab33b-positive vesicles contact FAs,Live-cell imaging of U2OS cells transfected with mCherry-Exoc6 (red), BFP-Rab33b (green), and vinculin-GFP (gray). The video shows magnification of the boxed area indicated in Figure 8a. Cells were imaged every minute using a spinning disk confocal microscope. Related to Figure 8.
