# Supplementary material for: Rab33b-exocyst interaction mediates localized secretion for focal adhesion turnover and cell migration
Source: iScience. 2022 Apr 14;25(5):104250. doi: 10.1016/j.isci.2022.104250 (PMC9061791; doi:10.1016/j.isci.2022.104250)
Supplement: Document S1. Figures S1–S5 and Table S1 [file mmc1.pdf]

iScience, Volume *25*

Supplemental information

Rab33b-exocyst interaction mediates localized secretion for focal adhesion turnover and cell migration

Synne Arstad Bjørnestad, Noemi Antonella Guadagno, Ingrid Kjos, and Cinzia Progida

Suppl. Figure 1

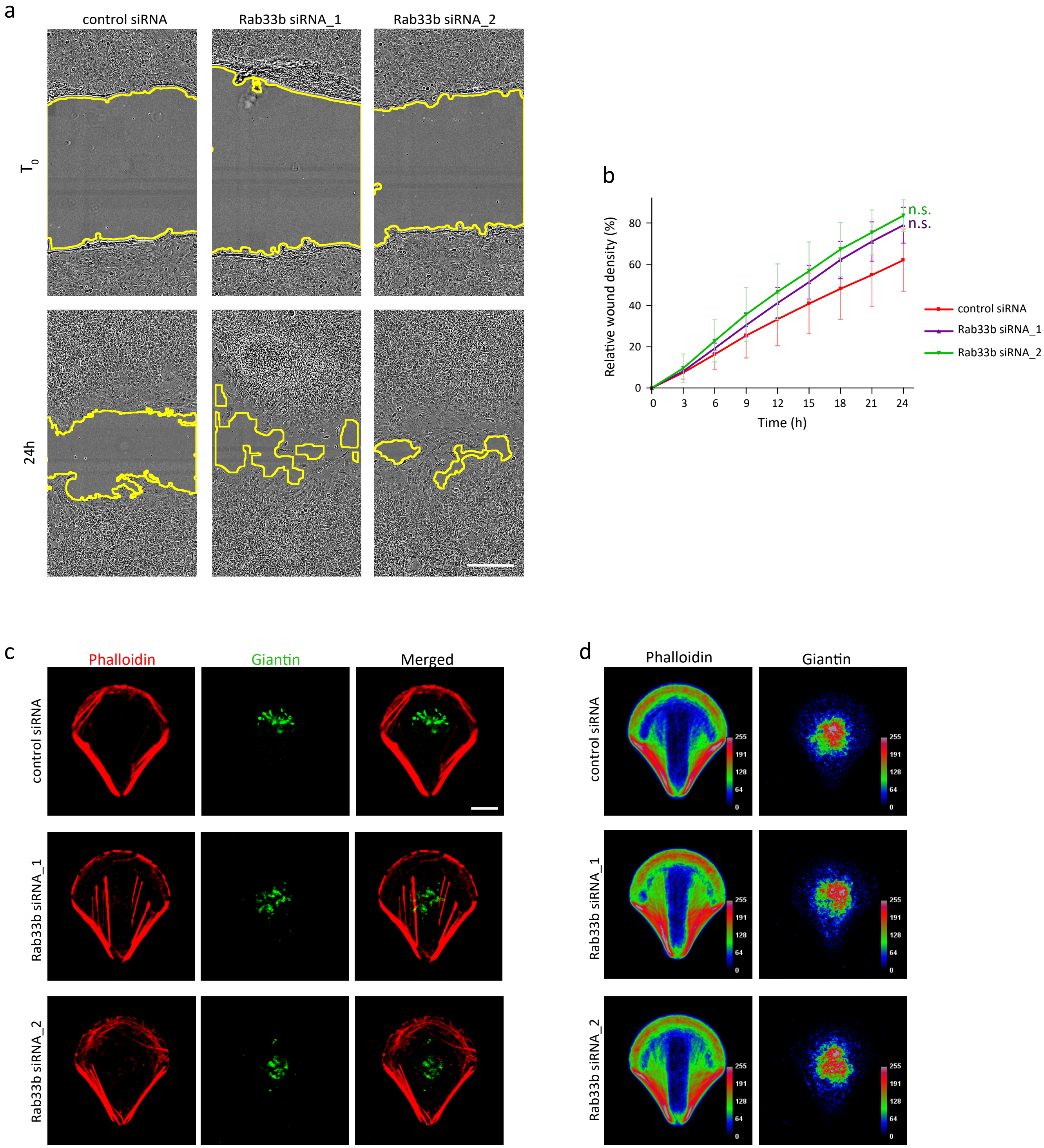

Suppl. Figure 2

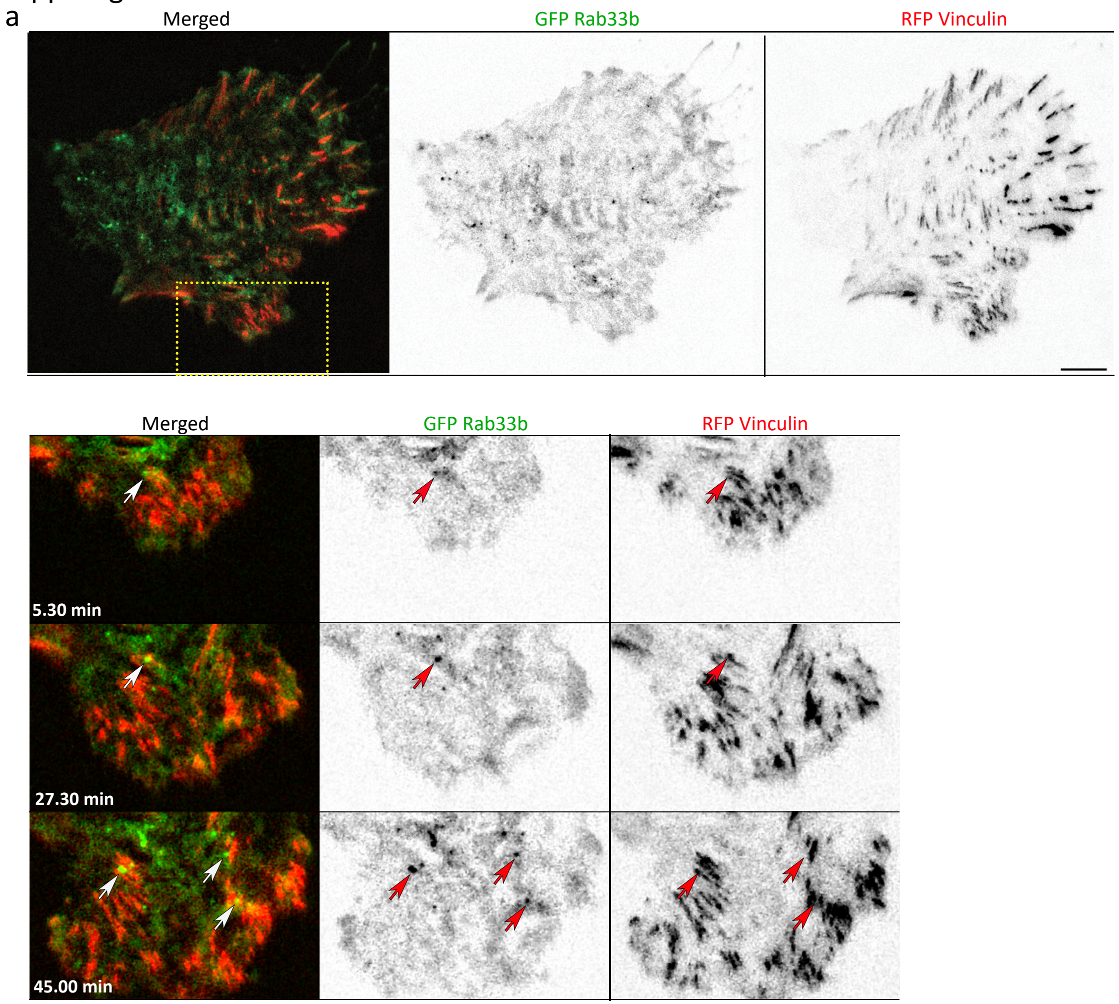

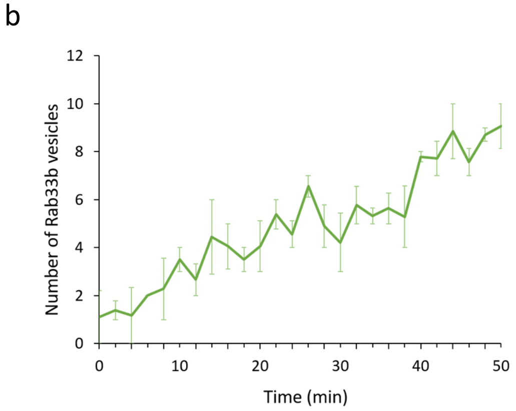

Suppl. Figure 3

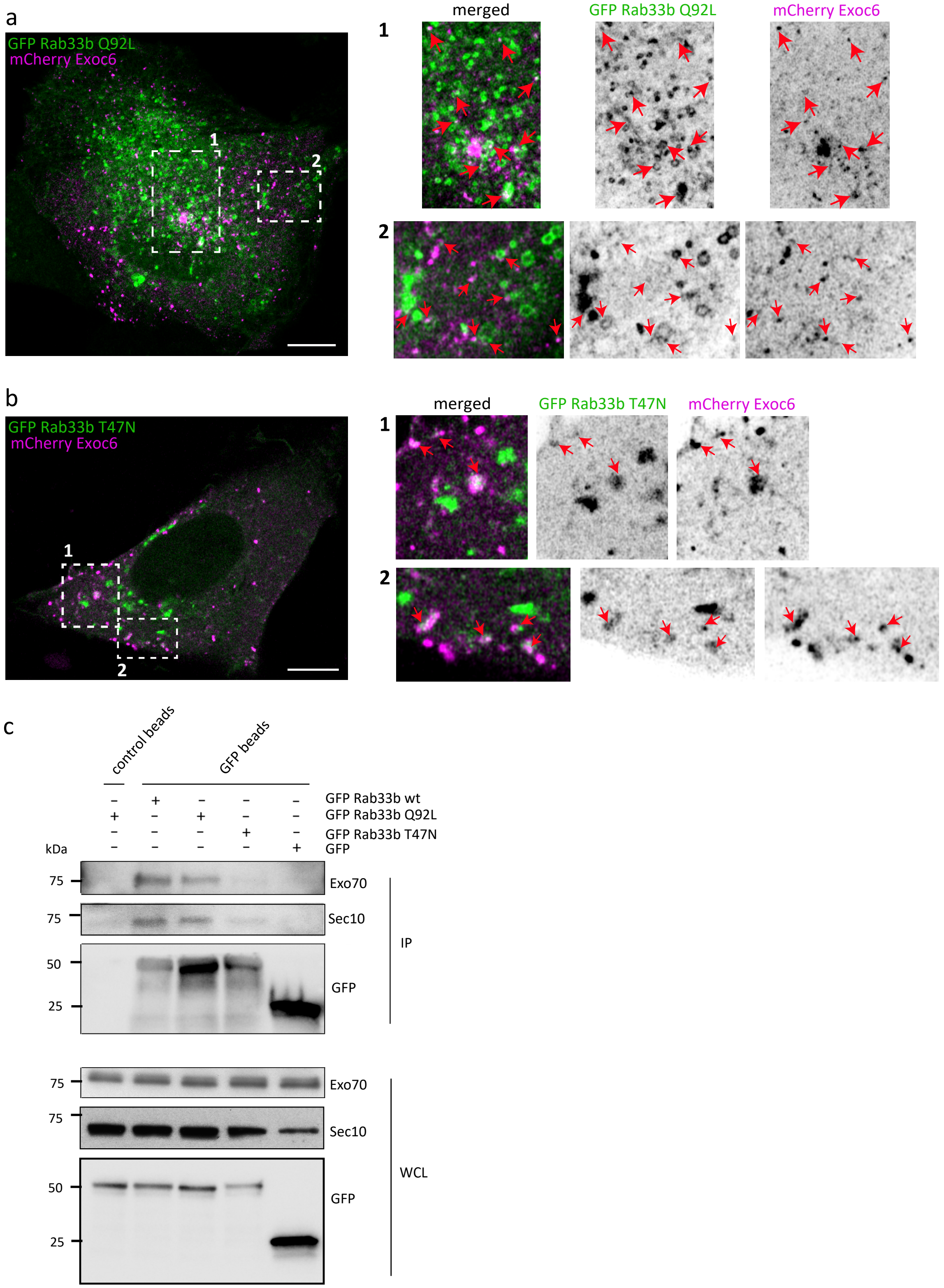

Suppl. Figure 4

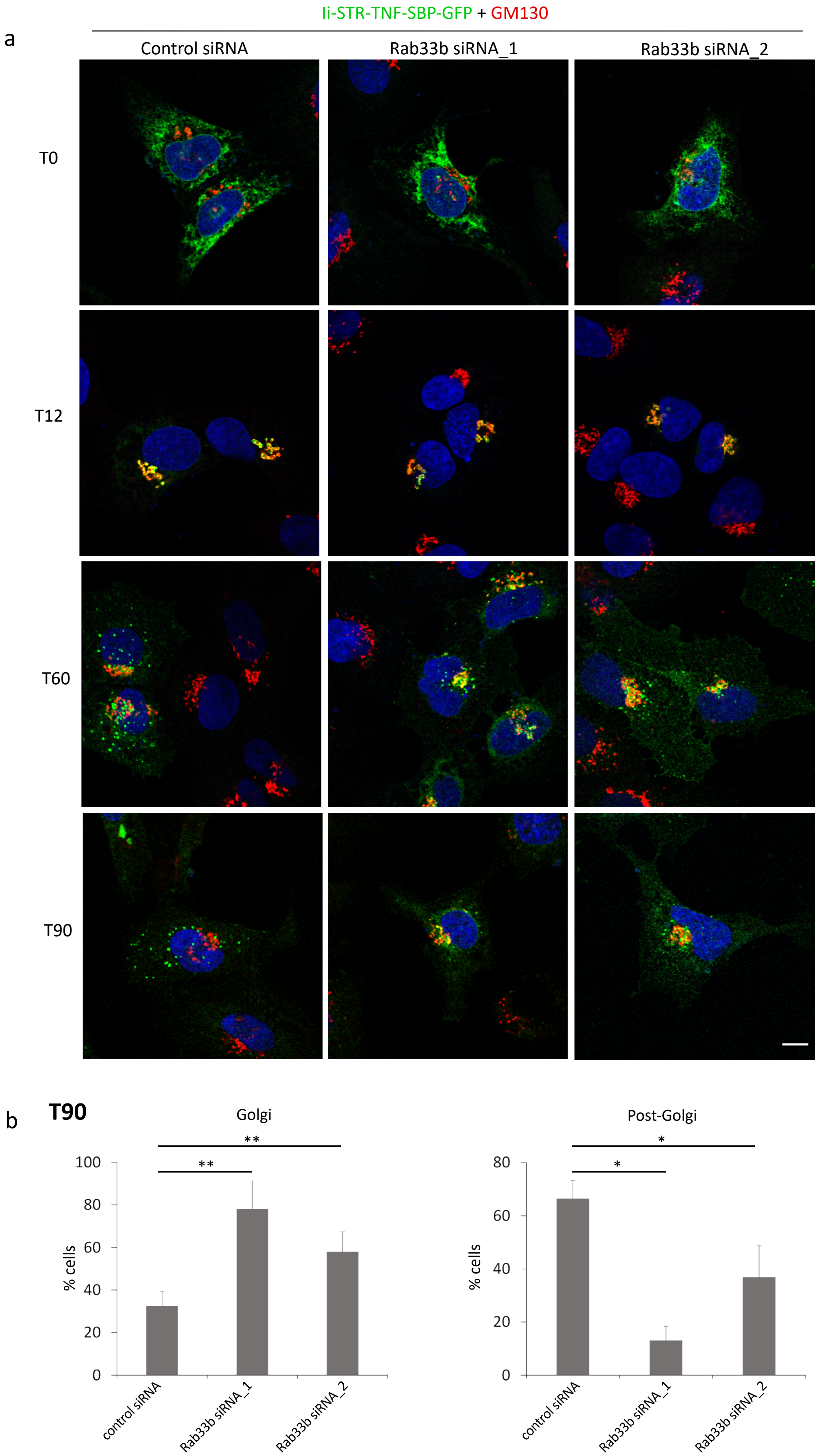

Suppl. Figure 5

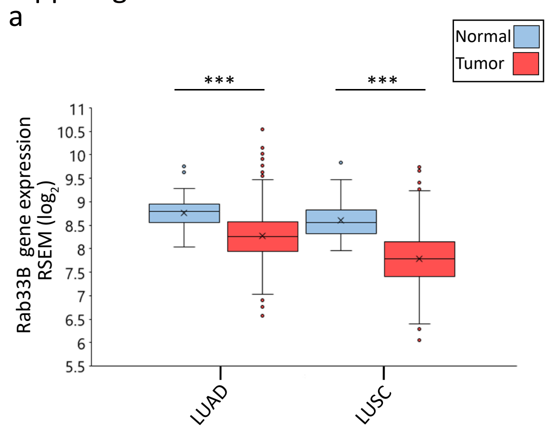

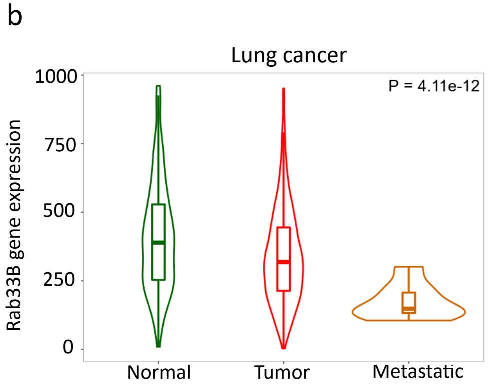

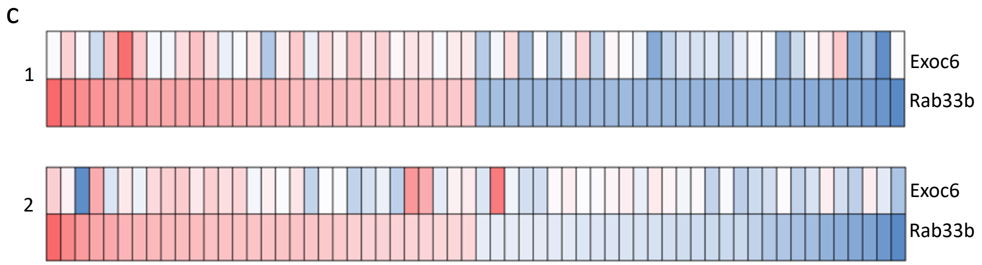

Supplementary figure legends

Supplementary figure 1. Silencing of Rab33b promotes cell migration but does not affect the Golgi apparatus, related to Figure 2 and 3.

(a) RPE-1 cells treated with siRNA control, Rab33b siRNA_1, Rab33b siRNA_2, were scratch-wounded and imaged every 15th minute for 24 h. Representative images of (T0) and 24 h after scratching are shown. Scale bar: 300 µm. (b) Graph showing relative wound density (%) for each sample in (a) over time. The graph represents the mean ± s.e.m of three independent experiments. (c) U2OS cells treated with siRNA control, Rab33b siRNA_1 or Rab33b siRNA_2, were plated onto fibronectin-coated crossbow-shaped micropatterns and left to adhere for 3.5 hours before fixation and staining with rhodamine-conjugated phalloidin and an antibody against giantin. Scale bar: 10 µm. (d) Color-coded map of the actin and giantin distribution for U2OS cells treated with siRNA control or Rab33b siRNAs. The images were obtained by using the CellRef macro on averaged Z-projection images from aligned single stacks.

Supplementary figure 2. Rab33b-positive vesicles are delivered to growing FAs during membrane protrusion, related to Figure 4.

(a) U2OS cells were transiently transfected with GFP-Rab33b and RFP-vinculin and imaged every 30 seconds using a TIRF microscope with a penetration depth of 90 nm. Magnifications of the boxed area show the formation of a protrusion over time. Arrows indicate Rab33b-positive vesicles in proximity of FAs. Scale bar: 10 µm. (b) The graph shows the number of Rab33b positive vesicles in forming protrusions over time. n = 10 cells from two independent experiments.

Supplementary figure 3. Rab33b interacts with the exocyst complex, related to Figure 5.

(a) Representative image of a U2OS cell co-transfected with GFP-Rab33b Q92L and mCherry-Exoc6. Red arrows in the magnifications of the boxed areas show colocalization of Rab33b Q92L (green) and Exoc6 (magenta) on vesicles. Scale bar: 10 µm. (b) Representative image of a U2OS cell co-transfected with GFP-Rab33b T47N and mCherry-Exoc6. Red arrows in the magnifications of the boxed areas show colocalization of Rab33b T47N (green) and Exoc6 (magenta) on vesicles. Scale bar: 10 µm. (c) U2OS cells were transiently transfected with GFP, GFP-Rab33b wt, GFP-Rab33b Q92L, or GFP-Rab33b T47N, lysed, and subjected to IP with GFP or control magnetic agarose beads. Whole-cell lysates (WCL) and immunoprecipitates (IP) were subjected to Western blot analysis using the indicated antibodies.

Supplementary figure 4. TNFα-SBP-EGFP transport to the cell surface is delayed by Rab33b depletion, related to Figure 7.

U2OS cells silenced with control siRNA, Rab33b siRNA_1 or Rab33b siRNA_2, and transiently transfected with Ii-Str-TNF-SBP-GFP (green) were fixed either immediately (T0), 12 minutes (T12), 60 minutes (T60) or 90 minutes (T90) after incubation with 40 µM D-biotin. Cells were then immunostained with an antibody against GM130. Scale bar: 10 µm. (b) Quantification of the TNFα distribution 90 minutes after the incubation with D-biotin. The percentage of cells in which TNFα had a total or partial overlap with the Golgi apparatus (Golgi) and no overlap with the Golgi apparatus (post-Golgi) was determined and represented as mean ± s.e.m. from four independent experiments. $n \geq 50$ cells. *$P < 0.05$, ** $P < 0.01$.

Supplementary figure 5. Expression of Rab33b in cancer, related to Figure 2 and 5.

(a) RSEM mRNASeq expression profiles of Rab33b in Lung adenocarcinoma (LUAD) and Lung squamous cell carcinoma (LUSC) vs. corresponding normal tissues from TCGA was evaluated by using FireBrowse.com. Student's two tailed homoscedastic t-test was used to analyze the statistical significance between Rab33b expressions in tumor vs. normal tissue. *** $P < 0.001$. (b) Rab33b gene expression in normal, tumor, and metastatic tissue from gene chip data at TNMplot.com. (c) Heat map of Rab33b and Exoc6 expression. mRNA expression (RNASeq V2 RSEM) datasets were obtained from the cBio Cancer Genomics Portal and the analysis indicates a positive correlation between Rab33b and Exoc6 expression in 1 = Lung Adenocarcinoma (TCGA, PanCancer Atlas), correlation $R = 0.534$, and 2 = Lung Adenocarcinoma (OncoSG, Nat Genet 2020), correlation $R = 0.293$.

Table S1: Overview of the siRNAs used in the siRNA primary screen, related to STAR Methods.
All gene symbols, gene IDs and siRNA catalogue number are indicated

Gene Symbol	Gene ID	siRNA catalogue number	Gene Symbol	Gene ID	siRNA catalogue number
control siRNA	-	D-001810-10	RAB21	23011	L-009450-00
RAB1A	5861	L-008283-00	RAB22A	57403	L-019214-00
RAB1B	81876	L-008958-01	RAB23	51715	L-009789-00
RAB2A	5862	L-010533-00	RAB24	53917	L-008828-01
RAB2B	84932	L-010370-02	RAB25	57111	L-010366-00
RAB3A	5864	L-009668-00	RAB26	25837	L-008793-00
RAB3B	5865	L-008825-00	RAB27A	5873	L-004667-00
RAB3C	115827	L-008520-00	RAB27B	5874	L-004228-00
RAB3D	9545	L-010822-00	RAB28	9364	L-008582-00
RAB4A	5867	L-008539-00	RABL2A	11159	L-013620-00
RAB4B	53916	L-008780-00	RABL2B	11158	L-008404-00
RAB5A	5868	L-004009-00	RAB30	27314	L-008269-00
RAB5B	5869	L-004010-00	RAB31	11031	L-010065-00
RAB5C	5878	L-004011-00	RAB32	10981	L-009920-00
RAB6A	5870	L-008975-00	RAB33A	9363	L-008206-00
RAB6B	51560	L-008548-00	RAB33B	83452	L-008909-00
RAB6C	84084	L-009031-00	RAB34	83871	L-009735-00
RAB7A	7879	L-010388-00	RAB35	11021	L-009781-00
RAB7B	338382	L-018225-00	RAB36	9609	L-009553-00
RAB8A	4218	L-003905-00	RAB37	326624	L-008933-01
RAB8B	51762	L-008744-00	RAB38	23682	L-010059-00
RAB9A	9367	L-004177-00	RAB39A	54734	L-026511-01
RAB9B	51209	L-010055-00	RAB39B	116442	L-008558-02
RAB10	10890	L-010823-00	RAB40A	142684	L-008924-00
RAB11A	8766	L-004726-00	RAB40AL	282808	L-184616-00
RAB11B	9230	L-004727-00	RAB40B	10966	L-008353-00
RAB12	201475	L-023375-02	RAB40C	57799	L-010368-00
RAB13	5872	L-008389-00	RAB41	347517	L-031575-01
RAB14	51552	L-009934-00	RAB42	115273	L-009144-02
RAB15	376267	L-031564-01	RAB43	339122	L-028161-01
RAB17	64284	L-006474-00	RAB44	401258	L-032182-02
RAB18	22931	L-010824-00	RASEF(RAB45)	158158	L-016768-01
RAB19	401409	L-028859-01	PLK1	5347	L-003290-00
RAB20	55647	L-008317-00			
